# A Phase I Double Blind, Placebo-Controlled, Randomized Study of the Safety and Immunogenicity of Electroporated HIV DNA with or without Interleukin 12 in Prime-Boost Combinations with an Ad35 HIV Vaccine in Healthy HIV-Seronegative African Adults

**DOI:** 10.1371/journal.pone.0134287

**Published:** 2015-08-07

**Authors:** Juliet Mpendo, Gaudensia Mutua, Julien Nyombayire, Rosine Ingabire, Annet Nanvubya, Omu Anzala, Etienne Karita, Peter Hayes, Jakub Kopycinski, Len Dally, Drew Hannaman, Michael A. Egan, John H. Eldridge, Kristen Syvertsen, Jennifer Lehrman, Beth Rasmussen, Jill Gilmour, Josephine H. Cox, Patricia E. Fast, Claudia Schmidt

**Affiliations:** 1 Uganda Virus Research Institute-IAVI, Entebbe, Uganda; 2 Kenya AIDS Vaccine Initiative, University of Nairobi, Nairobi, Kenya; 3 Projet San Francisco (PSF), Kigali, Rwanda; 4 IAVI, Human Immunology Laboratory, Imperial College, London, United Kingdom; 5 EMMES Corporation, Rockville, Maryland, United States of America; 6 ICHOR Medical Systems, Inc., San Diego, California, United States of America; 7 Profectus BioSciences, Inc., Tarrytown, New York, United States of America; 8 International AIDS Vaccine Initiative (IAVI), New York, New York, United States of America; Sanaria,. Inc, UNITED STATES

## Abstract

**Background:**

Strategies to enhance the immunogenicity of DNA vaccines in humans include i) co-administration of molecular adjuvants, ii) intramuscular administration followed by in vivo electroporation (IM/EP) and/or iii) boosting with a different vaccine. Combining these strategies provided protection of macaques challenged with SIV; this clinical trial was designed to mimic the vaccine regimen in the SIV study.

**Methods:**

Seventy five healthy, HIV-seronegative adults were enrolled into a phase 1, randomized, double-blind, placebo-controlled trial. Multi-antigenic HIV (HIVMAG) plasmid DNA (pDNA) vaccine alone or co-administered with pDNA encoding human Interleukin 12 (IL-12) (GENEVAX IL-12) given by IM/EP using the TriGrid Delivery System was tested in different prime-boost regimens with recombinant Ad35 HIV vaccine given IM.

**Results:**

All local reactions but one were mild or moderate. Systemic reactions and unsolicited adverse events including laboratory abnormalities did not differ between vaccine and placebo recipients. No serious adverse events (SAEs) were reported. T cell and antibody response rates after HIVMAG (x3) prime—Ad35 (x1) boost were independent of IL-12, while the magnitude of interferon gamma (IFN-γ) ELISPOT responses was highest after HIVMAG (x3) without IL-12. The quality and phenotype of T cell responses shown by intracellular cytokine staining (ICS) were similar between groups. Inhibition of HIV replication by autologous T cells was demonstrated after HIVMAG (x3) prime and was boosted after Ad35. HIV specific antibodies were detected only after Ad35 boost, although there was a priming effect with 3 doses of HIVMAG with or without IL-12. No anti-IL-12 antibodies were detected.

**Conclusion:**

The vaccines were safe, well tolerated and moderately immunogenic. Repeated administration IM/EP was well accepted. An adjuvant effect of co-administered plasmid IL-12 was not detected.

**Trial Registration:**

ClinicalTrials.gov NCT01496989

## Introduction

DNA vaccines have been extensively tested in humans and have shown a good safety profile but weak immunogenicity [1–4]. Since DNA vaccines offer a number of potential advantages over other vaccine approaches, ways to improve their immunogenicity continue to be investigated including: i) adjuvantation and/or ii) intramuscular (IM) or intradermal (ID) administration followed by *in vivo* electroporation (EP). One possibility for adjuvantation is co-administration with plasmids encoding cytokines such as Interleukin-12 (IL-12) [5, 6]. IL-12 plays a key role in the induction of adaptive immune responses and promotes cell-mediated immunity [7–9]. Delivery of DNA by electroporation has been shown to significantly improve immunogenicity compared to conventional injection [2, 4, 10–12]. The localized application of electrical fields is thought to lead to increased permeabilization of cell membranes which enhances the cellular uptake of large polar molecules such as DNA by a factor of 10–1,000 over conventional intramuscular injection [4]. In preclinical studies, delivery by *in vivo* EP has resulted in a greater magnitude of IFN-γ-producing T cells, greater proliferative capacity of CD8 T cells, and increased polyfunctionality of CD4 and CD8 T cells to various DNA vaccines [13, 14]. In humans, *in vivo* EP has been used to deliver DNA vaccines IM or ID, and intratumoral delivery has been used in cancer patients to administer vaccines or chemotherapeutic agents [15, 16]. A clinical trial in the USA of an HIV DNA vaccine in healthy volunteers showed that the response rate, magnitude, breadth and durability of the immune responses were significantly increased in the IM/EP group compared to the same DNA vaccine given by IM injection (response frequency of 88% IM/EP vs. 0% IM). Assessment of the tolerability indicated that the IM/EP procedure was acceptable for healthy, HIV-seronegative volunteers [17].

Two other studies have investigated different HIV DNA vaccines with or without plasmid IL-12 by IM/EP in US populations [18, 19]. Administration of an HIV DNA vaccine together with IL-12 (GENEVAX IL-12) by IM/EP (HVTN080) had a significant dose-sparing effect and provided CD4 and CD8 T cell responses superior to those observed in a previous trial (HVTN070) where the HIV DNA vaccine was given by standard needle injection [18, 19]. The HVTN087 trial tested Multi-antigenic HIV (HIVMAG at 3mg/dose x3) + IL-12 (GENEVAX IL-12) at 3 dosage levels (250μg, 1000μg, 1500μg) given by IM/EP using the TriGrid Delivery System (TDS) electroporation device followed by boosting with a vesicular stomatitis virus (VSV)-vectored Gag in 100 HIV-seronegative volunteers in the US. GENEVAX IL-12 at 1500ug increased the magnitude of CD8 T cell responses compared to no IL-12 [20].

Recombinant, replication defective, adenovirus type 35 (Ad35) constructs have previously been studied by IAVI and partners in 3 Phase 1 double-blind, randomized, placebo-controlled trials with a total enrolment of ~400 individuals, in the USA and Africa. The Ad35-GRIN (expressing a fusion protein composed of clade A Gag, RT, Integrase and Nef) administered alone or combined with Ad35-Env (expressing clade A gp140) induced predominantly CD8+ T cells recognizing all proteins as well as Env and Gag-p24 antibody responses [21, 22].

In a preclinical study, the whole SIVmac239 proteome was delivered to rhesus macaques (RM) in 5 separate SIV DNA plasmids by IM/EP with or without IL-12 pDNA followed by a boost with a corresponding Ad5-SIV vaccine. SIV DNA+IL-12 by IM/EP increased frequency and breadth across the proteome of Ag-specific CD4 and CD8 T cells and the production of multiple cytokines. There was no significant difference in the overall magnitude of SIV specific antibodies or CD8 T cell responses between groups, however, SIV+IL-12 by IM/EP induced a greater magnitude of SIV specific polyfunctional CD4 T cells than other regimens [23]. After repeated low dose SIVmac239 mucosal challenge, there was a significant log reduction of median SIV peak and set-point viral load, respectively, in RM vaccinated with SIV+IL12 by IM/EP compared to mock immunized controls (p <0.01). In 5 out of 6 infected RM, strong suppression of viremia was observed with intermittent ‘blips’ in virus replication. In 2 RM, no SIV RNA was detected in tissue and lymph nodes, even after 13 viral challenges. RM immunized without IL12 demonstrated a typical maximum of 1.5 log reduction in virus load [23].

The encouraging results in rhesus macaques warranted the testing of a similar vaccine regimen in humans. We hypothesized that i) adjuvanting HIVMAG with GENEVAX IL-12 would induce immune responses of greater magnitude, breadth and/or duration compared to HIVMAG alone, and ii) IM/EP delivery of cytokine-adjuvanted HIVMAG would induce robust and long lasting HIV-specific CD4+ and CD8+ responses and/or immune memory in a majority of vaccine recipients in prime-boost regimens with Ad35-GRIN/Env.

We report the results from a Phase I, double-blind, randomized, placebo-controlled study of pDNA HIVMAG +/- pDNA IL-12 (GENEVAX IL-12) and Ad35-GRIN/Env in five different prime-boost regimens, in which the dosage of IL-12, the number of HIVMAG administrations and the order of the two vaccines varied. This is the first study testing an adjuvanted HIV DNA vaccine given intramuscularly by IM/EP in healthy HIV-uninfected African adults at low-risk of HIV acquisition.

## Materials and Methods

### Ethics and Regulatory Approval

This study was approved by all respective local and international Ethics and Research Committees (ERCs), institutional biosafety committees, national regulatory agencies and the Recombinant DNA Advisory Committee (RAC)/NIH, USA. The study protocol was approved by the ethics committees of Kenyatta National Hospital, University of Nairobi, Kenya; the Uganda Virus Research Institute, Entebbe, Uganda and Projet San Francisco (PSF), Kigali, Rwanda, and reviewed by the responsible regulatory authorities in each country. Each study participant provided written informed consent prior to undertaking any study procedures. The study was conducted under BB IND# 14770 and in accordance with International Conference on Harmonization—Good Clinical Practice (ICH-GCP) and Good Clinical Laboratory Practice (GCLP).

### Study Design and Participants

This Phase 1 study of IL-12 plus HIVMAG was the first of its kind in Africa: it was designed to give a preliminary answer regarding safety and immunogenicity. The study was a multi-center, double-blind, randomized, placebo-controlled phase 1 trial of heterologous prime-boost regimens (Table 1). Eligible adults were recruited at clinical research centers (CRCs) in Uganda, Kenya and Rwanda using informational seminars. Volunteers were healthy, aged 18–50 years, at lower risk for HIV infection with confirmed negative serology for HIV-1 and HIV-2 infection, and willing to use an effective method of contraception. Pregnant or lactating women were not eligible. Volunteers with a clinically significant acute or chronic medical condition and volunteers carrying any electronic stimulation or other implantable device were excluded. Volunteer comprehension of the study was ascertained using an assessment of understanding tool. HIV prevention counseling was offered to all volunteers and HIV risk was assessed at multiple timepoints during the trial. Clinic and safety data collected at the CRCs were directly entered into an internet based data system on site. Immunogenicity data were uploaded to the data coordinating center (DCC, the EMMES Corporation) by the Human Immunology Laboratory (HIL).

**Table 1:** Study Design.

**Study Groups**	**N, vaccine/ Placebo**	**Months 0, 1, 2 (dosage, delivery)**	**Month 6 (dosage, delivery)**
**1**	**12/3**	HIVMAG* (IM/EP**)	Ad35-GRIN/Env***
**2**	**12/3**	HIVMAG + GENEVAX IL-12 (100μg) (IM/EP)	Ad35-GRIN/Env
**3**	**12/3**	HIVMAG + GENEVAX IL-12 (1000μg) (IM/EP)	Ad35-GRIN/Env
		**Month 0 (dosage, delivery)**	**Month 4 (dosage, delivery)**
**4**	**12/3**	HIVMAG + GENEVAX IL-12 (1000μg) (IM/EP)	Ad35-GRIN/Env
**5**	**12/3**	Ad35-GRIN/Env	HIVMAG + GENEVAX IL-12 (1000μg) (IM/EP)
**Total**	**75 (60/15)**		

*HIVMAG dose for all immunizations was 3mg

**IM/EP = intramuscular by electroporation

***Ad35-GRIN/Env was given at 2x10^10^ viral particles (vp), intramuscularly (IM)

### Study Vaccines

The HIVMAG vaccine—provided by Profectus BioSciences Inc.—consists of 2 DNA plasmids mixed at the time of administration. ProfectusVax HIV-1 clade B *gag/pol* + ProfectusVax HIV-1 *clade* B *nef/tat/vif*, *env (subtype B primary isolate Env gp160)*. HIVMAG was given at 3mg/dose by IM/EP. The HIV-1 MAG pDNA vaccine consists of 2 DNA expression vectors (WLV-151M and WLV-255M) encoding multiple HIV-1 clade B antigens [24]. The pDNA expression vector WLV-151M is a 7,487-bp plasmid expressing an HIV-1HXb2 Gag/Pol fusion protein under control of the human cytomegalovirus (HCMV) immediate early promoter and bovine growth hormone (BGH) polyadenylation (polyA) signal. The pDNA expression vector WLV-255M is a dual-promoter plasmid (8,750 bp) expressing a clade B primary isolate (HIV-6101) Env gp160 under control of the simian cytomegalovirus (SCMV) promoter and BGH polyA and an HIV-1NL43 Nef/Tat/Vif fusion protein utilizing the HCMV promoter and simian virus (SV) SV40 polyA tail. ProfectusVax DNA Plasmids were manufactured under cGMP by Boehringer Ingelheim Austria GmbH (BI Austria).

GENEVAX IL-12 is a recombinant DNA plasmid encoding p35 and p40 subunits of human IL-12 designed and manufactured as described previously [18, 19, 25]. Two dosage levels (100μg and 1000μg) were tested. IL-12 was mixed with HIVMAG immediately prior to injection and co-administered by IM/EP. Total injected volumes were 1.0mL, 1.3mL and 1.5mL for HIVMAG alone, HIVMAG + lower dosage of IL-12 and HIVMAG + higher dosage of IL-12, respectively. GENEVAX IL-12 was manufactured under cGMP by DSM Biologics (The Netherlands).

Both HIVMAG and IL-12 plasmids were formulated in 30 mM citrate buffer pH 6.5 containing 150 mM NaCl, 0.01% ethylenediamine tetraacetic acid (EDTA), and 0.25% bupivacaine-HCl [18, 19, 26].

The Ad35-GRIN and Ad35-Env are based on recombinant replication-defective adenovirus serotype 35 vectors. GRIN is a fusion protein composed of HIV -1 subtype A Gag, RT, Integrase, and Nef [21]. The vector encoding Env expresses HIV-1 clade A gp140. Ad35-GRIN and AD35-Env were co-formulated (Ad35-GRIN/Env) and administered as 0.5mL by intramuscular injection at 2x10^10^ viral particles (vp) per dose (1x10^10^ vp of GRIN and Env constructs). Ad35-GRIN and Ad35-Env were provided by IAVI. Ad35-GRIN vaccine was produced in HER96 cells by Transgene (Strasbourg, France) according to the principles of GMP.

Sodium Chloride, USP 0.9% was used as placebo and also as diluent for plasmid IL-12. The electroporation device used was the TriGrid Delivery System (TDS-IM) developed by Ichor Medical Systems, Inc. HIVMAG+/-IL-12 by IM/EP required bilateral administration at each vaccination timepoint with half the dose given into each deltoid muscle. Study physicians were specifically trained and qualified for IM/EP technique. Skin fold thickness was measured with a caliper at screening to determine penetration depth for the electrodes and the injection needle housed in the application cartridge.

### Outcomes

Primary endpoints including safety and tolerability of the different prime-boost regimens, secondary endpoints assessing the qualitative and quantitative immune responses elicited by the different prime-boost regimens and ancillary endpoints were pre-defined. (S1 File).

### Safety Assessments

Study participants were monitored by interim medical history, physical exam and laboratory assessments. Local (pain, tenderness, erythema/skin discoloration, swelling and induration) and systemic signs and symptoms (fever, chills, headache, nausea, vomiting, malaise, arthralgia and myalgia) were collected for 7 days after each vaccination. Volunteers were monitored at the clinic on day 0 (pre- and 30 minutes post-vaccination) and on days 3 and 7 after each vaccination. Participants were given 7-day diary cards and instructed to record the maximal signs and symptoms experienced each day. Unsolicited adverse events (AEs) were recorded through one month after the final vaccination and were graded for severity according to the Division of AIDS Adult Adverse Event Grading Toxicity Tables (Version 1.0, December 2004).

### EP Tolerability Questionnaire

Tolerability of the IM/EP procedure (assessed as pain and graded as none, light, uncomfortable, intense, severe and very severe) was monitored at 4 pre-determined timepoints (prior to, immediately after, 10 and 30 minutes after each IM/EP) using a standardized questionnaire which was administered in the clinic during the immediate post-injection observation period.

### Safety Monitoring

A Protocol Safety Review Team (PSRT), consisting of the principal investigators, study physicians, laboratory personnel, other clinic staff and the IAVI medical monitor, supervised the progress of the study and monitored safety data on an ongoing basis. An independent Safety Review Board (SRB) reviewed interim safety data at 2 pre-determined time points. Pause criteria were predefined in the study protocol (provided as S1 File).

### Randomization and Blinding

The randomization schedule was prepared by the data coordinating center (DCC), the EMMES Corporation. Volunteers were randomly assigned to one of five groups described in Table 1. Study site staff (except the pharmacist), volunteers, laboratory staff and medical monitors were blinded to assignment to vaccine or placebo and IL-12 dosage levels, but not to schedule and delivery method. Volunteers were randomized to vaccine or placebo in a 4:1 ratio, using a block size of 5, stratified according to site. Within each site volunteers were randomized in blocks of 5 (25 total), with each block consisting of one volunteer from each group, one of which was a placebo. The randomization list was provided to the site pharmacist of record for dispensing of vaccine and placebo. Investigators at the study sites enrolled volunteers via an electronic system (administered by the DCC), where allocation codes were assigned consecutively to eligible volunteers at the time of first vaccination.

### HIV Testing

The presence of vaccine–induced antibodies to HIV-1/2 using the Vironostika HIV-1/2 Ag/Ab EIA (bioMérieux, S.A., France) was assessed at baseline and while on study at pre-determined timepoints, both, after prime(s) and boost, respectively. Any positive HIV test result was followed up by HIV RNA PCR (Abbott m2000 Real Time PCR HIV-1 RNA kit, Abbott Park, IL) to distinguish vaccine-induced seroreactivity (VISR) from natural HIV infection. Pre- and post-HIV test counseling and individual risk reduction counseling were provided.

### Immunogenicity Assessments

#### Peripheral Blood Mononuclear Cell Sample Preparation

Peripheral blood mononuclear cells (PBMC) were isolated using density gradient separation from heparinized whole blood, frozen in a mixture of fetal bovine serum (Sigma-Aldrich, St Louis, MO, USA) and DMSO (90:10 ratio) using a Kryo 560–16 rate controlled freezer (Planer, Sunbury-On-Thames, UK). PBMC were stored and shipped in vapor phase liquid nitrogen to the IAVI Human Immunology Laboratory (HIL), Imperial College, London [21, 27].

#### IFN-γ ELISPOT Assay

Cellular immunogenicity was assessed by IFN-γ ELISPOT using frozen PBMC as previously described [21]. Peptide pools of 15-mer peptides overlapping by 11 amino acids were used at 1.5μg/mL; they were 90% pure by HPLC and covered the sequences of Clade B Gag, Pol, Nef-Tat-Vif and Env matched to the HIVMAG (JPT, Berlin, Germany) or Clade A gag, RT, Int, Nef and Env matched to Ad35-GRIN/Env (AnaSpec Inc, Fremont, CA, USA). A CMV pp65 peptide pool (quality control), PHA at 10μg/mL and a mock stimulus (DMSO/medium) were also used as previously described [21]. Spot forming cells (SFC) were counted using an automated AID ELISPOT reader (Autoimmun Diagnostika GmbH, Strassberg, Germany). A positive response was defined by the following criteria: 1) average number of background-subtracted spots in a single pool > specified cut-off of 38 SFC/10^6^ PBMC. The cut-offs were derived from assessing peptide pool responses in PBMC from at least 95 HIV seronegative individuals; 2) for each pool, if the number of replicates was 2 or ≥3, then the coefficient of variation (standard deviation/mean) between replicates had to be ≤ 50% or ≤ 70%, respectively; 3) mean count had to be >4 times mean background; 4) mean background had to be ≤ 55 SFC/10^6^ PBMC. Samples with mean background >55 SFC/10^6^ PBMC were considered failures and excluded from all analyses. For any subject, if pre-vaccination ELISPOT responses had a value greater than 38 SFC/10^6^ all subsequent responses to that peptide pool in that individual were considered cross-reactive and were not included in the frequency calculations.

#### Intracellular Cytokine Staining (ICS)

Flow cytometry was performed as described previously [21]. Antigen-specific phenotypes and cytokine secretion profiles were assessed using a qualified polychromatic flow cytometry (PFC) panel. PBMC were co-incubated with peptide pools matched to the HIVMAG and GRIN/Env inserts, 1 μg/ml SEB (Sigma-Aldrich, St. Louis, MO, USA) or mock stimuli, CD107a PECy5, BD Golgistop (Becton Dickinson, San Jose, CA, USA) and Brefeldin A (Sigma-Aldrich, Poole Dorset, UK) for 6 hours at 37°C. Cells were stained for viability with LIVE/DEAD Fixable Violet Dead Cell Stain Kit (Invitrogen, Eugene, OR, USA), and then surface stained by anti-CD4 QD605, anti-CD8 pacific orange, anti-CD19 pacific blue (Invitrogen, Paisley, UK), anti-CD27 APC-H7, anti-CD14 pacific blue, anti-CD57 FITC, anti-B7 integrin PE (Becton Dickinson, San Jose, CA, USA), and anti-CD45RO ECD (Beckman Coulter, High Wycombe, UK). Finally, intracellular staining was performed with anti-CD3 QD655 (Invitrogen, Paisley, UK), anti-IFN-γ PE Cy7, anti-TNF-α A700 and anti-IL-2 APC (Becton Dickinson, San Jose, CA, USA) washed and acquired on the same day. At least 750,000 events per sample were acquired on a custom-built BD LSR II cytometer. Data were analyzed and presented using FlowJo (version 8.8 Treestar, Ashland, OR, USA). ICS was performed on samples taken from Groups 1–3 only, at baseline, visits 07/08 (2–4 weeks post 3^rd^ HIVMAG prime) and visit 13 (2 weeks post Ad35 boost). From each of Groups 1–3, eight samples were picked for ICS analysis in a manner blinded to the laboratory staff consisting of 5 high ELISPOT responders, 2 low responders and 1 placebo.

#### Viral Inhibition Assay (VIA)

A VIA assay was qualified for use in vaccine trials as described below [28]. PBMCs were resuspended at a density of 1×10^6^ cells/mL in R10 medium supplemented with 50 U of IL-2 and 0.5μg/mL CD3/CD4 or CD3/CD8 bispecific antibodies (provided by Johnson Wong, Harvard Medical School) for generation of CD8 or CD4 T cells, respectively [29, 30]. Culture volumes were doubled at days 3 and 6 by addition of fresh medium and IL-2. CD4 T cells were infected, at a multiplicity of infection (MOI) of 0.01, for 3 h with a panel of 8 HIV-1 isolates—IIIB (subtype B), ELI (accession number A07108, subtype B), U455 (M62320 subtype A), and 97ZA012 (AF286227, subtype C) (provided by the HIV AIDS reagent repository), CH77 (FJ496000, subtype B), CH106 (NA, subtype B), 247FV2 (FJ496200, subtype C) (provided by George Shaw, University of Birmingham, AL, USA) and CBL4 (subtype D, kindly provided by the National Institutes of Biological Standards and Control, UK). Supernatant p24 content was measured on day 13 by enzyme-linked immunosorbent assay (ELISA) (PerkinElmer, Waltham, MA, USA). CD8+ T cell-mediated inhibition was expressed as the log_10_ reduction in p24 content of day 13 CD8+ and CD4+ T cell co-cultures, compared with infected CD4+ T cells alone. For clinical trial volunteers, antibody-expanded pre-vaccination CD4+ T cells were used as common targets for HIV-1 infection in co-cultures with pre- and post-vaccination CD8+ T cells. The threshold used for positive inhibition was determined from previous validation studies as reduction in measurable p24 production of >1.5 logs. VIA was assessed on Groups 1–3 at baseline, 2 or 4 weeks post 3^rd^ HIVMAG, and 2 weeks post Ad35-GRIN/Env vaccine.

#### HIV-specific Binding Antibodies

An ELISA assay was used to measure HIV specific Env and Gag antibody responses at baseline and at indicated times post-vaccination. End-point titration of serum was performed in 96-well medium binding plates (Greiner Bio-one, Frickenhausen, Germany) coated with preparations of 2.5μg/mL purified recombinant subtype B Gag p24 (Aalto Bio Reagents Ltd., Dublin, Ireland), 5μg/mL subtype A Env UG037 gp140 protein (81% matched to the Ad35-Env insert) made by Polymun Scientific Immunbiologische Forschung, Vienna, Austria or 5μg/mL subtype B Env 6101 gp140 protein (exactly matched to the HIV-MAG Env WLV255M insert). Titers were determined by sequential incubation of antigen with serum followed by HRP-labeled anti-human IgG and TMB (3, 59, 5, 59-tetramethyl-benzidine) substrate. After addition of stop solution, the optical density (OD) at 450 nm was measured for 5-fold serially diluted samples starting at 1/100. The titer was calculated as the most dilute serum concentration above the OD cut off of 0.3 and 0.2 respectively for Env and Gag p24 and reported as reciprocal dilution.

#### Anti-IL-12 Antibody Assay

Assays were performed by Profectus BioSciences Inc., Tarrytown, NY, USA using serum from baseline and 4 weeks post final dose of HIVMAG in Groups 1–5. The IL-12 neutralization assay uses a natural killer (NK) cell line, NK-92MI. The NK-92MI is a human NK cell line that shows a dose dependent secretion of IFN-γ in response to hIL-12. Neutralizing antibodies that block hIL-12 biological function would reduce IFN-γ secretion by NK-92MI cells. The IFN-γ secreted in the culture supernatant was quantified in a sandwich ELISA using commercial reagents and an IL-12 positive control. The lower limit of quantification for this assay was determined to be 8 neutralization units/mL (NU/mL) and titers equal to or less than 8 NU/mL were assigned a value of 4 NU/mL. The cutoff for positive responses (31.5 NU/ml) was calculated as the 97.5^th^ percentile of all baseline observations (n = 75). Baseline titers were positive if greater than 31.5 NU/ml, whereas post-vaccination titers were positive if greater than 31.5 NU/ml or greater than twice baseline if the baseline assay was positive.

### Statistical Methods

Once the sample size of 75 (60 vaccine and 15 placebo) across five arms was determined, the power calculations and detectable effect sizes were based on the sample size. Fisher’s exact test (for categorical variables) and Kruskal-Wallis Test (for continuous variables) were used to compare the balance and/or values of baseline characteristics between the study Groups. All safety and immunogenicity comparisons were made using Fisher’s exact, 2-tailed tests of the proportions of volunteers with an endpoint, unless otherwise stated. The safety comparisons were based on the maximum severity per volunteer. All tests are 2-tailed; statistical significance was defined as a p <0.05, unadjusted for multiple comparisons except for pairwise comparisons among the study groups, where p <0.01 was considered to be statistically significant. Analyses were performed using SAS version 9.2, (SAS, Cary, NC, USA). Immunogenicity results were summarized within each group at each time-point using descriptive statistics for continuous variables and percentages (with 95% CI) for categorical variables.

## Results

### Participant Flow and Recruitment

**Fig 1:**
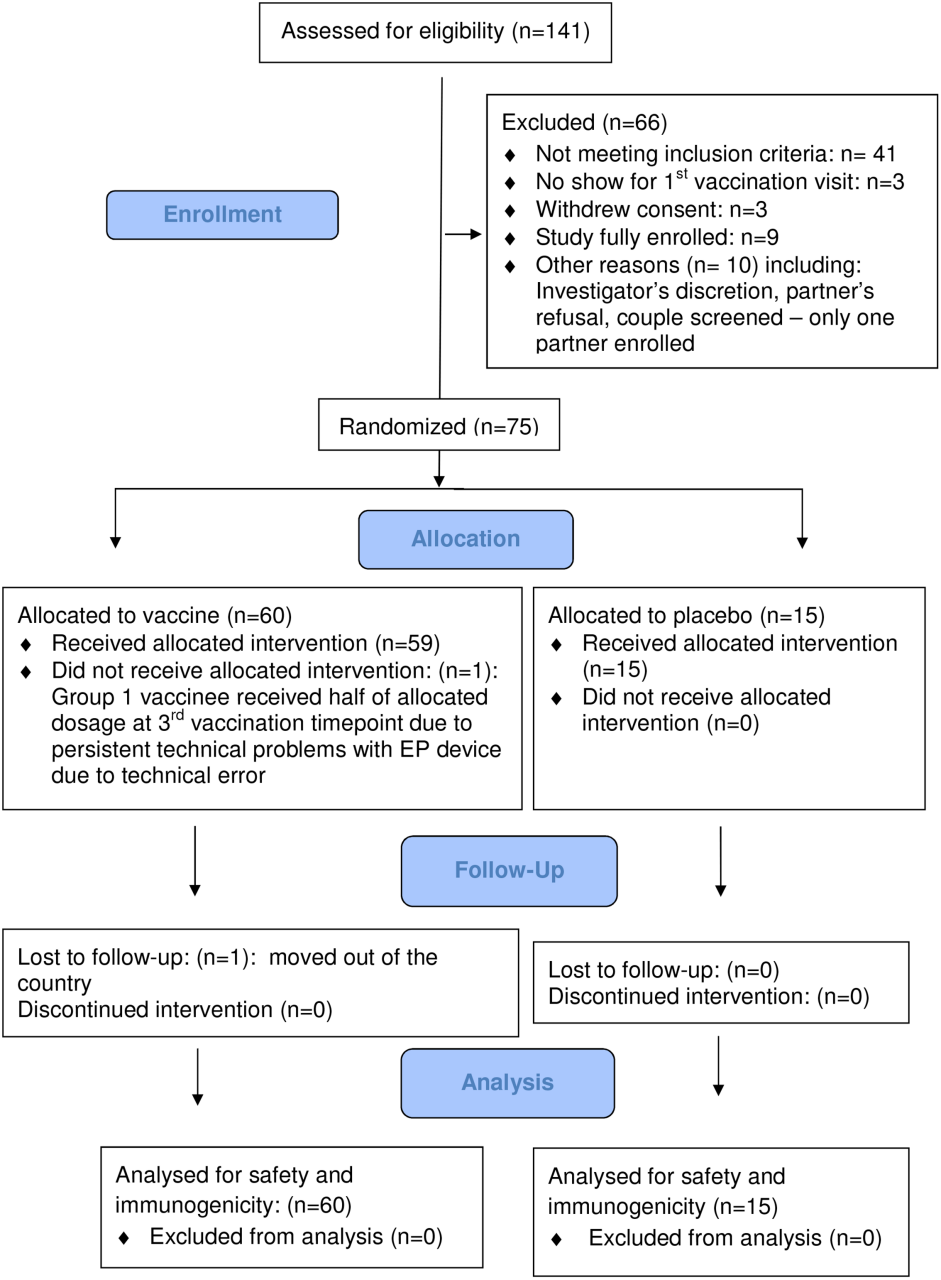
CONSORT Diagram.

A total of 141 adults were screened, 75 healthy, HIV-seronegative adults (29 [39%] females) were enrolled, 25 at each of the 3 clinical research centers (CRC). Screening started on 13 December 2011, the first enrolment occurred on 19 December 2011, the last enrolment on 24 April 2012, and the last follow-up on 26 March 2013. (Fig 1: **CONSORT Diagram**). Table 2 describes the baseline demographic characteristics of the enrolled volunteers. Participants reported low risk for HIV (i.e. sexual abstinence, or two or fewer mutually monogamous relationships with partners who did not use illicit drugs, or two or fewer partners believed to be HIV-uninfected who did not use illicit drugs and with whom he/she regularly used condoms for vaginal and anal intercourse) within 12 months before enrollment. Sexually active female participants agreed not to become pregnant and to use effective contraceptive methods at least until 4 months after the final vaccination. All study participants except one completed all vaccinations as per protocol. One Group 1 vaccinee received only half the intended dose of HIVMAG alone at the 3^rd^ administration timepoint due to persistent technical problems with the EP device. None of the pause criteria was met.

**Table 2:** Baseline Demographics.

	DEMOGRAPHICS: Number and (%)						
	Group 1	Group 2	Group 3	Group 4	Group 5	Placebo	Total
Number of Volunteers	12	12	12	12	12	15	75
Sex							
Female	4 (33%)	7 (58%)	4 (33%)	5 (42%)	4 (33%)	5 (33%)	29 (39%)
Male	8 (67%)	5 (42%)	8 (67%)	7 (58%)	8 (67%)	10 (67%)	46 (61%)
Race							
Black African	12 (100%)	12 (100%)	12 (100%)	12 (100%)	12 (100%)	15 (100%)	75 (100%)
Age (yrs)							
Mean	30.2	30.6	30.2	32.6	30.1	29.7	30.5
Range	21–41	23–45	22–46	23–46	21–43	21–45	21–46
Vaccinations Received							
Vaccination #1,2	12 (100%)	12 (100%)	12 (100%)	12 (100%)	12 (100%)	15 (100%)	75 (100%)
Vaccination #3,4	12 (100%)	12 (100%)	12 (100%)	na	na	9 (100%)	45 (100%)

### Vaccine Safety

**Fig 2:**
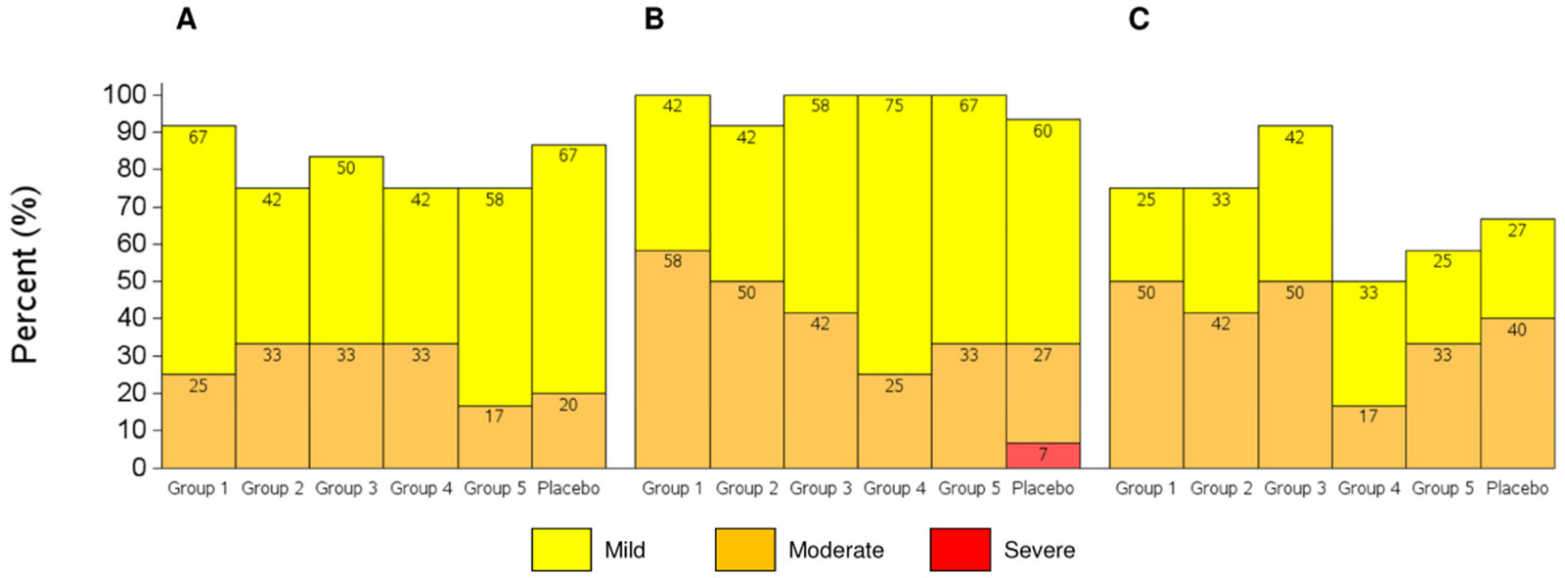
Maximum Severity of Adverse Events (AE). **A**. Local Solicited AE over 7 days, **B**. Systemic Solicited AE over 7 days, **C**. Unsolicited AE over 28 days.

The overall frequency of any local or systemic reaction or any AE was not statistically significantly different in vaccine compared to placebo recipients and was also independent of receipt of plasmid IL12. Most volunteers reported reactogenicity events during the 7-day post-vaccination period, but all systemic and all local events were mild or moderate in severity, except for one case of severe local tenderness in a placebo recipient following a second attempt of IM/EP administration due to a technical error with the EP device (Fig 2A, 2B and 2C, S1 Table). There were no reports of severe or greater unsolicited AE’s (including hematology or biochemistry laboratory parameters), no SAEs, no intercurrent HIV-infection, no discontinuation of vaccination due to an AE and no pregnancies in the post-vaccination period when contraception was required (S3 File). No event met pausing criteria.

### Vaccine-induced Seroreactivity (VISR)

At the final study visit, 21/60 (35%) vaccine recipients tested HIV positive by Vironostika HIV1/2 Ag/Ab test without being HIV infected. The percentage of vaccine recipients with vaccine-induced seroreactivity (VISR) was 50% in Group 1, 42% each in Groups 2 and 3, 8% in Group 4 and 33% in Group 5.

### Electroporation Device Malfunctions

A total of three hundred thirty (330) injections (HIVMAG+/- IL-12 or Placebo) were administered IM/EP using the TDS-IM. Device malfunctions or technical errors were reported at 15 vaccinations in a total of 12 volunteers. All errors were appropriately managed and all except one volunteer received their vaccinations and dosages as per protocol.

### Electroporation Tolerability

Describing the maximum tolerability rating overall for all groups combined (3 IM/EP administrations in groups 1–3 and 1 IM/EP administration in groups 4–5), the majority of volunteers reported some discomfort, but no volunteers rated discomfort as severe or very severe at any time point. S2 Table summarizes the maximum rating per volunteer (vaccine and placebo recipients) over all tolerability assessment time points. Overall, discomfort was greatest immediately after the electrical stimulation, but improved rapidly. S1 Fig shows the maximum tolerability rating per volunteer in placebo and vaccine recipients in groups 1 to 5, by time point. There was no report of intense pain in any Group 1 (no IL-12) volunteer at any timepoint. At the time of IM/EP administration, almost all vaccine recipients in Group 1 (92%) rated the procedure as uncomfortable while 54% of placebo recipients and 33%, 75%, 50% and 50% of vaccine recipients in Groups 2–5, respectively, rated it as uncomfortable or intense (combined). There was no statistical significant difference in proportions of volunteers who rated the procedure as either uncomfortable or intense at any timepoint among the 6 groups (5 vaccine and 1 placebo, range 42% to 67%).

Multiple logistic regression models were fit to investigate the possible effects of age, gender, BMI, skin-fold thickness and dose group (no IL-12, 100μg IL-12, 1000μg IL-12 and placebo) on the probability of rating the procedure as uncomfortable or intense at each time point after each vaccination. At the first and second IM/EP administrations the odds of the procedure being uncomfortable or intense were statistically significantly lower in older volunteers and in those with lower skin-fold thickness (both at various time points), higher in volunteers with lower BMI (at the time of IM/EP administration), and higher in females than males (30 minutes after the first administration)–data not shown. For the third administration no significant effects were found and there was no effect of IL-12 dosage at any time. The multivariate proportional odds regression analyses of tolerance is shown in S3 Table. Almost all volunteers said yes when asked whether IM/EP would be an acceptable mode of administration if it helped protect against serious disease (96% of volunteers) or provided additional scientific knowledge (97%).

### Cellular Immune Responses

#### IFN-γ ELISPOT; Frequency of Response

**Fig 3:**
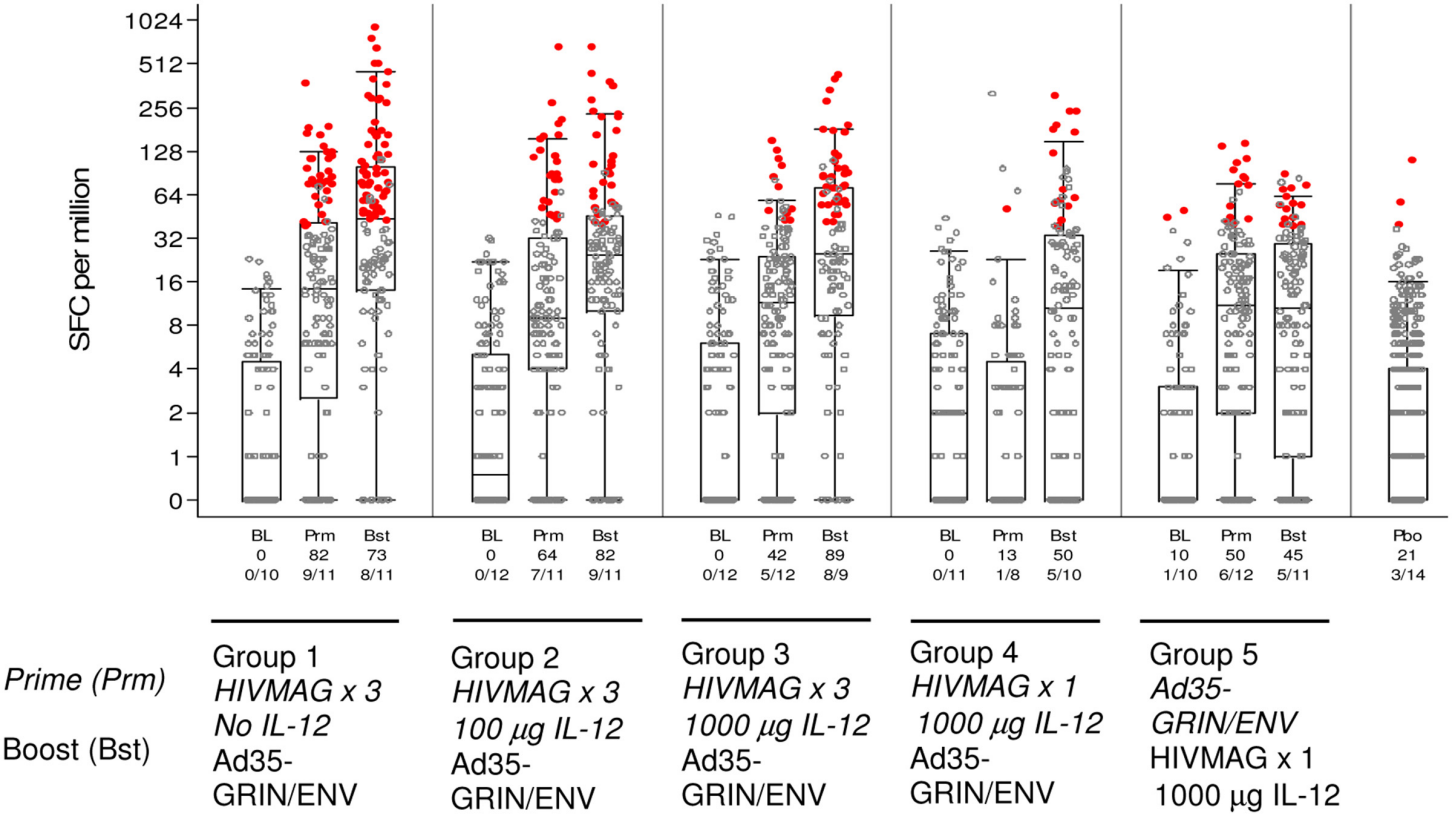
Total IFN-γ ELISPOT Response Rates and Magnitude. Responses at baseline (BL), 2 weeks after *prime (Prm)* and 2 weeks after boost (Bst) are shown. The y-axis shows the SFC/10^6^ PBMC on a half-log scale. All responses are background-subtracted. Mean responses <1 were set to 1. Black dots: response below the cut-off as defined in the materials and methods to any of the 12 MAG and GRIN-Env peptide pools; red circles: response above the cut-off to any of the 12 peptide pools. For the vaccine groups, the overlaid box plot summarizes the positive responses (i.e., the median, 1st and 3rd quartiles and minimum/maximum). The x-axis shows the % responders and number of responders out of the total tested at each time point. The placebo (Pbo) responses are combined in the far right box plot.

The percentage of participants who had an IFN-γ ELISPOT response to any peptide pool 2 weeks after HIVMAG (x3) was 82%, 64% and 42% in volunteers who received no plasmid IL-12, 100μg, or 1000μg IL-12 in Groups 1–3 respectively (not significantly different). Two weeks after Ad35-GRIN/Env boost, the response rates were 73%, 82% and 89% (Fig 3) with no statistically significant differences noted. After priming with HIVMAG (x3), response rates to the 6 HIVMAG matched peptide pools were seen more frequently than to the GRIN/Env matched peptide pools and after the boost, response rates to both sets of peptide pools were seen with similar frequency (data not shown). The response rates were 13% two weeks after a single injection of HIVMAG, and 50% two weeks after the Ad35-GRIN/Env boost (Group 4). When the order of the vaccines was reversed (Group 5), 50% responded after Ad35-GRIN/Env ‘prime’ and 45% had a positive ELISPOT response after HIVMAG (x1) boost. The response rate to any peptide pool (any HIVMAG or GRIN/Env peptide pool) in Group 1 HIVMAG (x3) followed by Ad35-GRIN/Env after all vaccinations showed a trend to higher response rate (73%) than those seen in Groups 4 and 5 (50 and 45% respectively). At 4 weeks post final vaccination response rates were about the same as those at 2 weeks post final vaccination. Responses to any of the HIVMAG or GRIN/Env antigens were durable to the last study visit, when positive response rates were 63%, 78% and 50% at week 24 post-boost in Groups 1–3, respectively, 50% and 25% at 32 weeks post-boost in Groups 4–5 respectively (data not shown). Overall at 2 weeks after the last vaccination, there were no significant differences in the response rate and magnitude of the ELISPOT response between groups 1–3, however some significant differences were noted between groups 1 and 2 and groups 4 and 5 (S4 Table). There were no positive responses at baseline for any volunteer except one in Group 5 who had a response to one peptide pool.

#### IFN-γ ELISPOT; Magnitude of Response

ELISPOT magnitude is shown as the sum of the positive responses for each volunteer over the HIV-MAG or GRIN/Env peptide pools (Table 3). In volunteers with a positive ELISPOT response to any of the 6 HIVMAG peptide pools, the geometric mean of the sum of the responses to all peptide pools at 2 weeks post three HIVMAG administrations was similar across Groups 1–3 (227, 234 and 135 SFC/m, respectively). In contrast, there were no positive ELISPOT responses to any of the 6 HIVMAG peptide pools 2 weeks after a single administration of HIVMAG in Group 4. Following the Ad35 GRIN/Env boost, the geometric mean of the sum of the 6 GRIN/Env peptide pools was highest in group 1 (418 SFC/m PBMCs) and similar across groups 2–4 (142, 203 and 227 SFC/m, respectively). By comparison, the geometric mean ELISPOT response across the 6 GRIN/Env peptide pools was 117 SFC/m in Group 5 following a single Ad35 without priming, thus demonstrating that HIVMAG primed the response to Ad35 GRIN/ENV. A single dose of HIVMAG did not boost a response primed by Ad35-GRIN/ENV (Group 5).

**Table 3:** HIV-specific IFN-γ ELISPOT Responses, Magnitude and Breadth after HIVMAG +/-IL-12 and Ad35 GRIN/Env.

	HIV-MAG peptide pools*					GRIN-Env peptide pools*				
Group	N/Total	Priming Regimen	Mean (SE)	GM (95% CI)	Breadth	N/Total	Boosting Regimen	Mean (SE)	GM (95% CI)	Breadth
1	8/11	HIVMAG (x3)	337 (114)	227 (102–506)	3.0	8/11	Ad35 GRIN/Env (x1)	620 (172)	418 (171–1025)	3.6
2	7/11	HIVMAG + 100μg IL-12 (x3)	391 (141)	234 (78–706)	2.9	8/11	Ad35 GRIN/Env (x1)	172 (41)	142 (82–246)	1.4
3	5/12	HIVMAG + 1000μg IL-12 (x3)	155 (35)	135 (61–299)	1.8	8/9	Ad35 GRIN/Env (x1)	269 (72)	203 (102–403)	2.1
4	0/8	HIVMAG + 1000μg IL-12 (x1)	---	---	---	4/10	Ad35 GRIN/Env (x1)	253 (56)	227 (91–569)	1.8
5	6/12	Ad35 GRIN/Env (x1)	149 (41)	117 (50–273)	2.0	3/11	HIVMAG + 1000μg IL-12 (x1)	129 (51)	110 (19–630)	2.3

*Sum of all background-subtracted positive responses for each volunteer over the 6 HIV-MAG or GRIN/Env peptide pools. N = Number of positive assays. GM = Geometric Mean. CI = Confidence Interval. SE = Standard Error. Breadth = Mean number of positive peptide pools per volunteer.

#### IFN-γ ELISPOT; Breadth of Response

**Fig 4:**
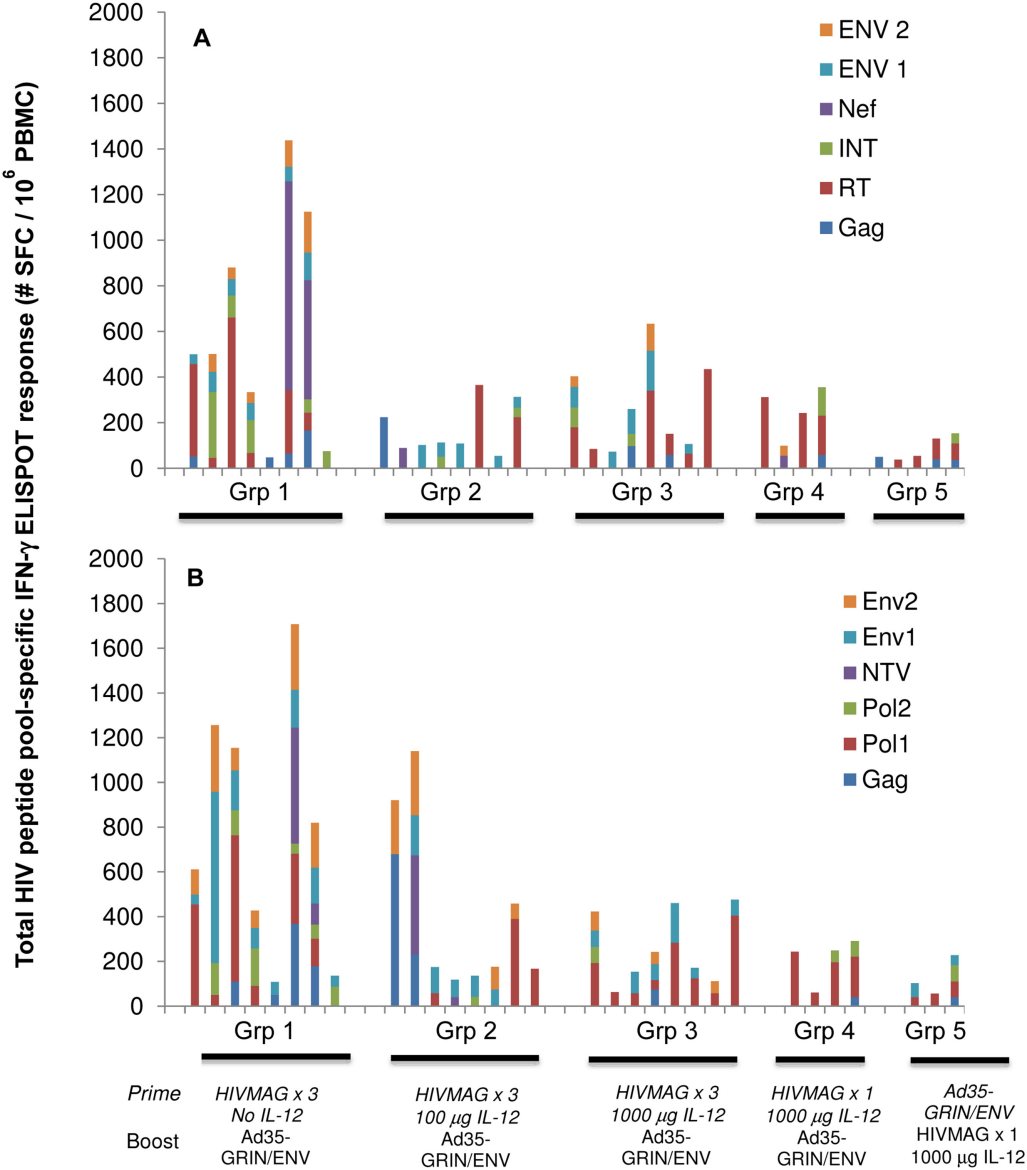
HIV-specific IFN-γ ELISPOT responses in HIVMAG and Ad35-GRIN/Env immunized individuals. At 2 weeks post final immunization in each of groups 1–5, PBMCs were collected and tested for GRIN/Env (**A**) and HIVMAG (**B**) specific IFN-γ secretion by ELISpot assay. Each stacked bar represents a response from an individual volunteer with each color representing a different peptide pool. Only individual peptide pool-specific responses that met the criteria for positivity are included.

Plasmid IL-12 did not increase the breadth of the ELISPOT response to HIVMAG, nor prime for a broader response to Ad35 GRIN/Env. In Groups 1–3 volunteers with a positive ELISPOT response to any of the 6 HIVMAG peptides, the average number of pools recognized post HIVMAG (x3) was 3, 2.9 and 1.8, respectively, (Table 3) and after Ad35-GRIN/Env boost, the average number of GRIN/Env pools recognized was 3.6, 1.4 and 2.1, respectively. Overall, after prime and boost, the most frequently recognized HIVMAG and GRIN/Env peptide pools were Env and Pol-RT peptides (Fig 4).

#### Intracellular Cytokine Staining (ICS)

**Fig 5:**
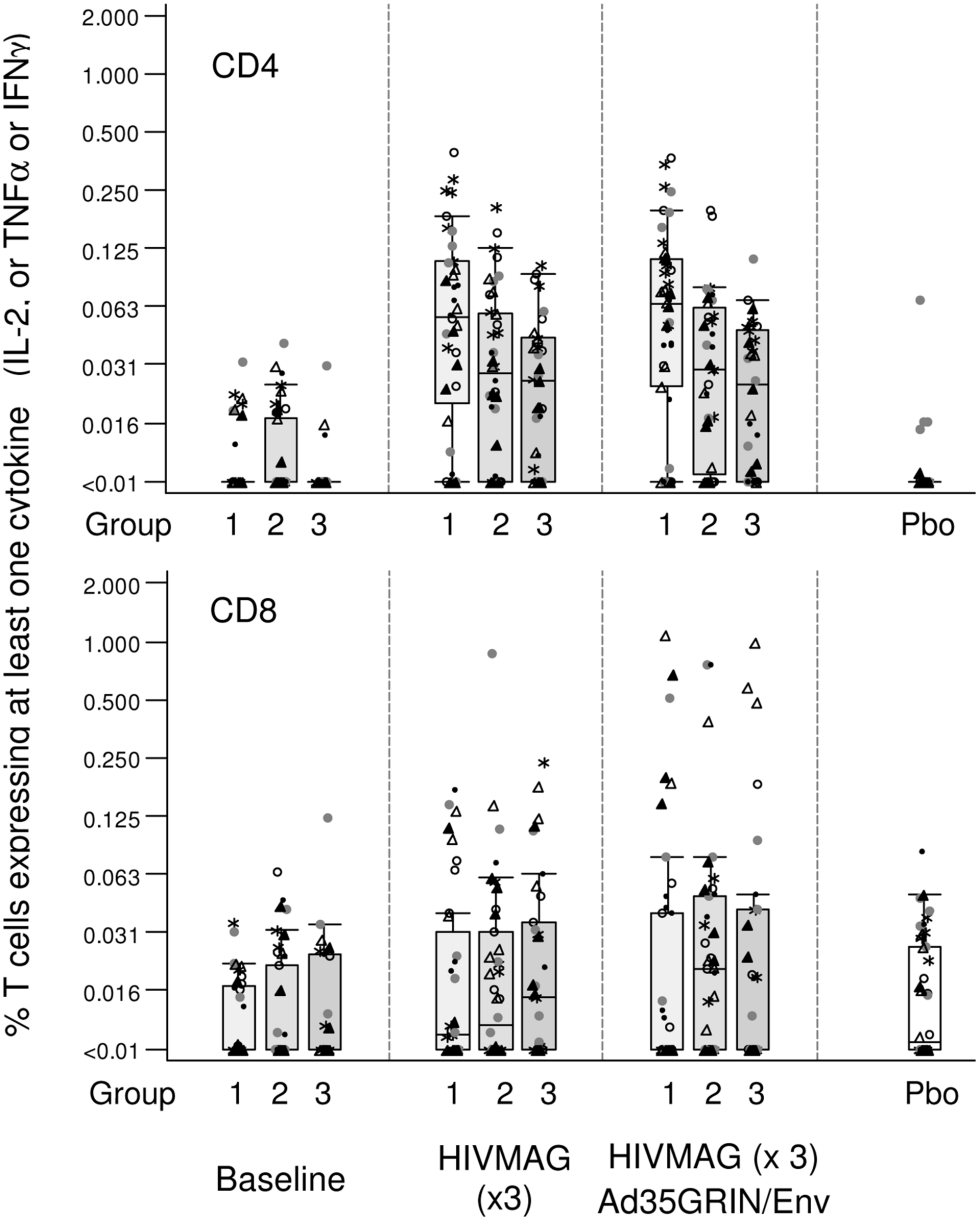
CD8+ and CD4+ T-lymphocyte Responses by Intracellular Cytokine Staining and Polychromatic Flow Cytometry. ICS was performed on a subset of samples from Groups 1–3, at baseline, visits 07/08 (2–4 weeks post HIVMAG (x3) prime) and visit 13 (2 weeks post Ad35-GRIN/Env boost). HIV-1-specific CD4+ (top panel) and CD8+ (bottom panel) T cell responses were evaluated by a 7-color ICS to assess the expression of IL-2, IFN-γ and TNF-α with peptides matched to the HIVMAG. Symbols show responses to MAG peptide pools; Env pool 1 = *, Env pool 2 = ○, Gag = ●, Nef Tat Vif = ●, Pol pool 1 = Δ, Pol pool 2 = ▲. The y-axis shows the % expressing T cells on a half-log scale.

In order to assess the quality of the cellular immune response in vaccinated individuals, a 7-color panel was used to look at cytokine staining in CD4+ and CD8+ T cells at three time points in Groups 1–3 using the HIV-MAG peptide pools (S2 Fig). Eight samples were picked in a manner blinded to the laboratory staff consisting of 5 high and 2 low ELISPOT responders and 1 placebo from each of Groups 1–3. Fig 5 (top panel) shows that HIV-specific CD4 T cell responses were elicited after the HIV-MAG vaccinations with a range up to 0.5% of T cells expressing at least one cytokine amongst IL-2, TNF-α and IFN-γ and a trend for fewer T cell responses in the IL-12 arms compared with Group 1 that received no IL-12. Specifically, in vaccine recipients at 2 weeks after HIVMAG (x3), the median percent of CD4+ T cells expressing at least one cytokine amongst IL-2, TNF-α and IFN-γ were 0.055, 0.028, and 0.026% in Groups 1–3, respectively. At 2 weeks after the Ad35-GRIN/Env boost, median CD4 responses were 0.065, 0.029 and 0.025% in Groups 1–3, respectively (Fig 5, top panel). HIV-specific CD8 T cell responses were elicited after 3 doses of HIVMAG but with a lower magnitude compared to CD4 responses and responses were similar across Groups 1–3. The median percent of CD8+ T cells expressing at least one cytokine was 0.011, 0.012 and 0.015% in Groups 1–3, respectively after 3 doses of HIVMAG. Two weeks after the Ad35-GRIN/Env boost, the median percent of CD8+ T cells expressing at least one cytokine was 0.003, 0.021 and 0.007% in Groups 1–3, respectively, with a range up to 1% (Fig 5, bottom panel). As shown with the ELISPOT assay, T cell responses were to multiple HIVMAG proteins. The polyfunctionality of CD4+ or CD8+ T cells expressing one, two or three cytokines (IFN-γ, IL-2 or TNF-α) to any HIVMAG matched was also assessed. The profile of CD4 and CD8 polyfunctionality was similar across Group 1–3, with a trend for greater CD4+ T cell polyfunctionality after HIVMAG (x3) prime and for greater CD8+ T cell polyfunctionality after Ad35-GRIN/Env (data not shown).

#### Viral Inhibition Assay (VIA)

**Fig 6:**
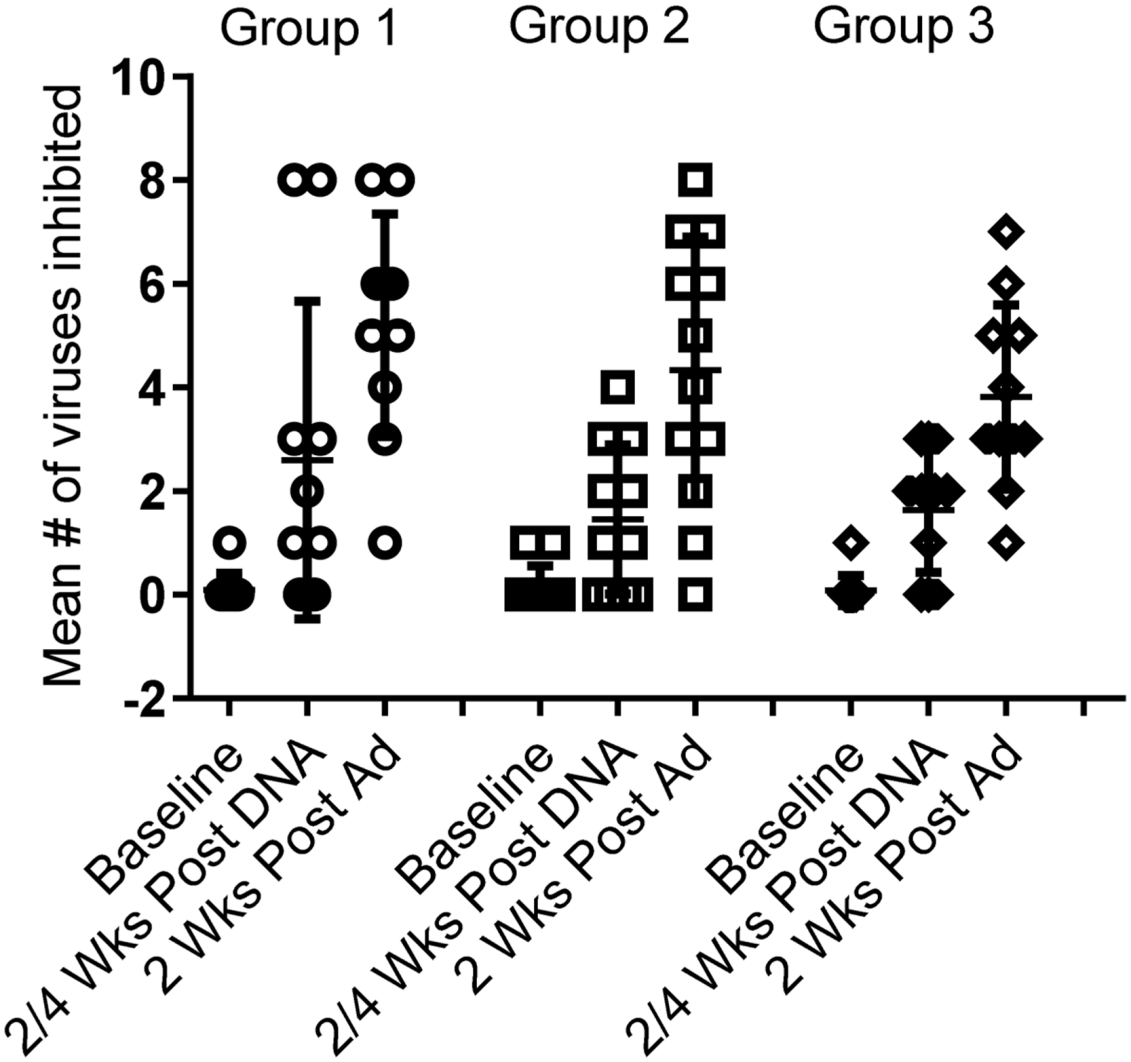
VIA breadth. VIA was assessed at baseline, 2–4 weeks post HIVMAG (x3) and at 2 weeks post Ad35-GRIN/Env boost in Groups 1–3. Each symbol represents an individual and the bar shows the mean number of viruses inhibited out of a total of 8 viruses in the panel.

Viral inhibition against a panel of 8 viruses was assessed for Groups 1–3. After HIVMAG, 73%, 64% and 73% of individuals, respectively, had VIA activity to one or more of the 8 viruses in the panel. After Ad35-GRIN/Env boost, 100%, 92% and 100% respectively in Groups 1–3 had VIA activity to at least one virus (Table 4). There was no significant difference in VIA magnitude (S3 Fig) or response rate among Groups 1–3 vaccine groups (p = 0.18). None of the five placebo recipients had any VIA activity at any time and one each of the 7 volunteers in Groups 2 and 3 had weakly positive VIA at baseline to one of the 8 viruses. The mean breadth was 2.6, 1.5 and 1.6 of eight viruses inhibited after the HIVMAG (x3) prime and 5.2, 4.3 and 3.8 recognized after the Ad35-GRIN/Env boost in Groups 1–3, respectively. The presence or dose of plasmid IL-12 did not affect breadth across groups 1–3 after prime or boost (Fig 6).

**Table 4:** Distribution of Log Inhibition Responses and Percent Positive Responses in each Group to any Virus.

	Placebo*		Group 1**		Group 2**		Group 3**	
	Prime	Boost	Prime	Boost	Prime	Boost	Prime	Boost
# Volunteers	5	5	11	11	11	12	11	11
Median Log Inhibition***	0.50	0.69	1.0	2.2	0.8	1.8	0.87	1.5
Maximum Log Inhibition***	1.34	1.30	4.28	4.66	4.23	4.80	3.30	4.23
VIA responders	0/5	0/5	8/11	11/11	7/11	11/12	8/11	11/11
% Response rate	0	0	73	100	64	92	73	100

*Groups 1–3 placebos combined

** Group 1; HIVMAG prime, Ad35 GRIN/Env boost, Group 2; HIVMAG + 100ug IL-12 prime, Ad35 GRIN/Env boost, Group 3; HIVMAG + 1000ug IL-12 prime, Ad35 GRIN/Env boost

***Each volunteer had results for 8 viruses and the median and maximum log inhibition is based on all 8 results and all volunteers per group.

### Humoral Immune Responses

**Fig 7:**
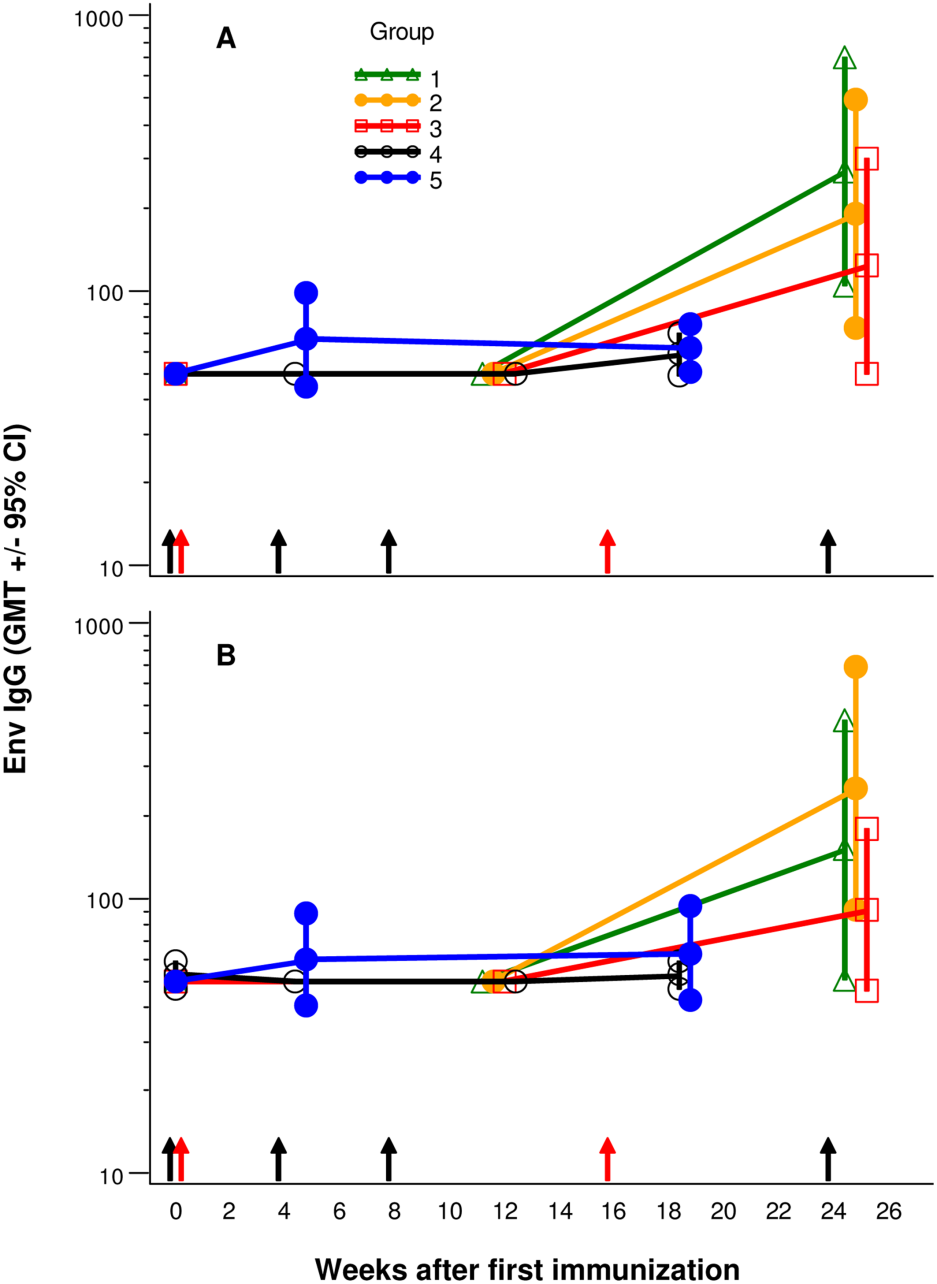
Env Antibody Titers. Panel A) HIV-Env subtype A (UG37) and panel B) HIV-Env subtype B (6101) specific IgG antibody titers were assessed at baseline, post prime and post boost. Black and Red arrows indicate vaccination times for Groups 1–3 and Groups 4–5, respectively.

Env subtype A, Env subtype B and Gag specific antibody titers and response rates are shown in S5 Table. Env subtype A and B antibody titers across groups 1–5 are shown in Fig 7. In Groups 1–3 no positive subtype A UG37 Env antibody titers were observed after priming with HIVMAG (x3) +/- plasmid IL-12, however, 2 weeks after the Ad35-GRIN/Env boost, there was a robust response; response rates were 89%, 89% and 67%, respectively. Similarly in Group 4 no positive titers were observed after HIVMAG (x1) prime, but after the Ad35 boost, positive titers were observed in 25% of samples. In Group 5, positive titers were observed in 25% of vaccinees 4 weeks after a single Ad35-GRIN/Env prime injection; this response rate was not improved by HIVMAG+IL-12 (x1) boost. Comparison of the frequency of responders to in group 5 (Ad35-GRIN/ENV) with Groups 1–3 showed that the priming with HIVMAG+/-IL-12 has a significant effect. There was an overall significant difference in UG37 Env antibody response rates between groups 1, 2, 3 and 5 (Fisher exact 2-tail test p = 0.0053). When comparing Group1 or Group 2 vs Group 5 there was a significant difference in response rates, (p = 0.0075), but there was no significant difference in response rate for Group 3 vs Group 5. Moreover, comparison of the responder frequency in group 4 with groups 1–3 showed that 3 priming doses of the DNA-EP is superior to one. The differences between Groups 1, 2 and 3 were not significant. All baseline and placebo titers were negative.

Antibody responses to the subtype B Env 6101 (EnvB), measured by ELISA, showed a similar pattern to that seen with the subtype A Env, though of lower magnitude and response rate (S5 Table and Fig 7). Antibody responses to subtype B Gag p24 were sporadic and of low titer (S5 Table).

### Anti-IL-12 Antibodies

At baseline, 2 samples had anti-IL-12 neutralizing antibodies above the threshold for positivity (one in a vaccine recipient (Group 3) and one in a placebo recipient), but there were no positive responses at any post-vaccine visits (data not shown).

## Discussion

This was the first clinical trial conducted in an African population, evaluating vaccine administration by IM/EP and the effect of plasmid IL-12 as an HIV DNA vaccine adjuvant. Participants were also given Ad35 GRIN/Env, as boost or, in one group, as prime before the DNA vaccine. The vaccines and regimens tested were safe and well tolerated, which is consistent with results from other clinical studies testing HIV DNA+IL-12 pDNA and Ad35-GRIN/Env in the US [18, 19, 21]. *In vivo* electroporation was well accepted, also consistent with reports from other clinical trials using the same or a different IM/EP device [17–19]. The pain reported prior to the IM/EP procedure likely reflects anxiety rather than actual pain. No volunteer refused subsequent IM/EP administrations. The majority of participants indicated acceptance of IM/EP vaccination for a pandemic infectious disease such as HIV.

The vaccines and regimens in this study were immunogenic, producing IFN-γ ELISPOT responses in ~80% of all vaccinated individuals, although the magnitude of response to each of the 12 individual peptide pools was relatively modest compared to other Ad35-GRIN-Env prime-boost regimens (unpublished data from IAVI) and Keefer et al [21]. ICS responses assessed in a few representative individuals in Groups 1–3 were likewise modest in magnitude, with responses skewed to CD4 after the HIVMAG prime and toward CD8 after the Ad35-GRIN/Env boost. Responses to DNA delivered by IM/EP were surprisingly strong compared to responses reported in other trials for DNA given by simple IM injection [31–33].

Anti-HIV inhibitory capacity is considered to be an important attribute of CD8+ T cells and such activities may help control HIV-viral load during acute and chronic infection particularly in long-term non-progressors [34–38]. In the present trial, viral inhibition activity (VIA) was noted after HIVMAG (x3) in Groups 1–3, which has not previously been observed after DNA vaccination with or without EP [17, 31]. After the Ad35-GRIN/Env boost, VIA magnitude, breadth and response rates were increased significantly compared to the DNA prime; plasmid IL12 did not seem to have an effect, as the responses were similar across Groups 1–3, both before and after boosting. Overall, VIA magnitude, response rate and breadth were similar to that seen in other HIV vaccine trials using a similar assay but of lower magnitude than in long-term non-progressors [21, 22, 31]. CD4+ cell polyfunctionality, as detected by IFN-γ, IL-2 and TNF-α expression was similar across Groups 1–3. Overall T cell responses assessed by ELISPOT, ICS and VIA were not enhanced by IL-12 administration. Although there were some trends to indicate a negative effect of IL-12 on immune responses, the differences did not reach statistical significance.

In two previous clinical trials with HIV DNA vaccines, the use of IM/EP increased response rates considerably and/or had dose sparing effects compared to DNA given by conventional intramuscular injection [17, 19]. Two dosage levels of plasmid IL-12 were selected for this dose-ranging study based on data from previous clinical trials, in which up to 1500μg IL-12 have been tested. Human data on the effect of IL-12 as an adjuvant for HIV DNA vaccines, however, are limited and results have been inconsistent [7, 18, 19, 39, 40]. In our trial the effects of IM/EP on cellular immunogenicity could not be assessed, as there was no group where HIVMAG+/-IL-12 was given by standard IM injection. Overall, these results did not demonstrate an adjuvant effect of plasmid IL-12 at the doses used in this EP/DNA vaccine.

Reports of the adjuvant effect of IL-12 in preclinical models are also inconsistent, with some effect being shown in some but not other preclinical models. A dose ranging study in mice demonstrated a bell shaped curve for adjuvant activity of IL-12 on T cell responses after a single injection of IL-12 at dosage levels of 0.1, 0.3, 1.0, 3.0, 10.0 and 30.0μg when co-administered with HIV gag DNA at 5 and 25μg/ dose (Egan, M et al unpublished). Studies in non-human primates showed significant increase in cell-mediated and humoral immune responses in the groups that received SIV DNA+IL-12 compared to SIV DNA alone [23, 25, 41, 42]. In the preclinical study, which set the stage for this clinical study, there was no significant difference in the overall magnitude of SIV specific antibodies or CD8 T cell responses between groups, however, SIV+IL-12 by IM/EP induced a greater magnitude of SIV specific polyfunctional CD4 T cells than other regimens. After repeated low-dose SIVmac239 mucosal challenge, a significant log reduction of the median SIV set point viral loads was seen in rhesus macaques that received SIV DNA+IL-12 by EP compared to the median set point viral loads in the absence of IL-12 [23]. One study showed that rhesus IL-12 (1.5 mg and 5.0 mg) augmented humoral and cellular immune responses to SIV gag DNA compared to DNA with no IL-12, but there was no difference between the low and high IL-12 dosage level [25]. Finally, in clinical studies IL-12 therapy for cancer has shown some clinical benefits in some trials but the potential of IL-12 as an adjuvant or therapeutic has not been fully realized to date [7, 39, 40].

Env binding antibodies were not detected after one or more doses of HIVMAG prime, but were detected after Ad35-GRIN/Env prime or boost similar to previous studies [31, 33]. The lack of antibody responses after DNA vaccination with HIVMAG is similar to the findings from other clinical trials testing HIV DNA+/-IL-12 [18, 19]. However, there was some evidence of an anamnestic response for elicitation of antibodies, since the GMT and response rates in Groups 1–3 were 2–3 times higher than those in group 5 after a single Ad35-GRIN/Env administration. Similar anamnestic effects of DNA for antibody responses have been seen in other studies where a DNA vaccine was followed by an Ad5 boost [31, 33].

One of the scientific rationales for this study was to see whether DNA prime including plasmid IL-12 increased the quantity and quality of HIV-specific CD4 T cells because these changes appeared to be correlated with increased protection from pathogenic SIVmac239 in the pre-clinical study of rhesus macaques immunized with SIV DNA + IL-12 by IM/EP [23]. In this clinical study we were not able to detect improved magnitude of response or polyfunctionality of HIV-specific CD4 in the presence of IL-12. Differences in the SIV DNA and Ad5 constructs, use of an optimized rhesus IL-12 [42], multiple injections sites (simultaneous injections into each limb), different IM/EP device, different species and other factors in the NHP study may account for this apparent discrepancy in the effect on CD4 cells. It should be noted that we also do not have a clear understanding of the role of EP DNA IL12 and enhanced CD4+ responses on the mechanism of protection in the NHP experiment.

In summary, HIVMAG DNA vaccine with or without IL-12 in combination with Ad35-GRIN/Env was safe, well tolerated and moderately immunogenic; the regimen induced CD4 and CD8 T cells and antibodies; the CD8 T cells could inhibit HIV replication in autologous CD4 T cells. Synergy between the priming with DNA and boosting with Ad35-GRIN-/ENV were demonstrable for antibody and cell inhibitory responses. Repeated administration by IM/EP was acceptable among African volunteers. Based on the excellent safety profile and volunteer acceptance of the EP procedure in Africa as well as a desire to better understand the effect of EP on immune responses, another study was designed. This study ‘Safety & Immunogenicity of HIV Vaccines in Healthy Kenyan Adults (HIV-CORE 004, NCT02099994)’ is currently ongoing in Nairobi, Kenya. Seventy two volunteers have been enrolled, vaccinations have been completed and immunogenicity assessments are ongoing.

## Supporting Information

S1 File Protocol. A Phase I double blind, randomized, placebo-controlled, study of safety and immunogenicity of a multi-antigenic HIV-1 DNA vaccine given intramuscularly by electroporation followed or preceded by multigenic Adenovirus subtype 35 vector vaccine in healthy HIV uninfected African adults.
(PDF)

S2 File CONSORT checklist. (DOC)

S3 File Supplementary Safety Information. (DOCX)

S1 Table Summary of Adverse Events within 28 Days of any Vaccination: by Relationship, Severity and Study Group. (DOCX)

S2 Table Summary of Electroporation Tolerability Assessments Overall. (DOCX)

S3 Table Multivariate proportional odds regression analyses of tolerance. (DOCX)

S4 Table Pair-wise comparisons of ELISPOT responses 2 weeks after the final vaccination. (DOCX)

S5 Table Env and Gag ELISA data. (DOCX)

S1 Fig Maximum tolerability assessment per volunteer over all EP administrations received. (DOCX)

S2 Fig ICS Gating. (DOCX)

S3 Fig VIA magnitude across groups to panel of 8 viruses. (DOCX)
