# Supplementary material for: A Phase I Double Blind, Placebo-Controlled, Randomized Study of the Safety and Immunogenicity of Electroporated HIV DNA with or without Interleukin 12 in Prime-Boost Combinations with an Ad35 HIV Vaccine in Healthy HIV-Seronegative African Adults
Source: PLoS One. 2015 Aug 7;10(8):e0134287. doi: 10.1371/journal.pone.0134287 (PMC4529153; doi:10.1371/journal.pone.0134287)
Supplement: S3 File — (DOCX) [file pone.0134287.s003.docx]

**Supplementary Safety Information:**

**Local reactogenicity**

Overall, local reactions were reported by 98.3% of vaccine and 93.4% of placebo recipients, and moderate or severe (n=1) local reactions were reported by 41.7% vaccine and 33.4% placebo recipients, respectively (Chi-Square test; p=0.556). The most common local reactions were pain and tenderness; a placebo recipient reported severe tenderness following an electroporation device failure during the first vaccination. Local reactions were self-limited and resolved within 1-3 days. Severity of reactions did not increase with following vaccinations in any group. Erythema and induration at the injection site were rare and were less severe than Grade 1 as per DAIDS Toxicity Tables (data not shown).

**Systemic reactogenicity**

Overall, systemic reactions were reported by 80.0% of vaccine and 86.7% of placebo recipients, and moderate systemic reactions were reported by 28.3% of vaccine and 20.0% of placebo recipients (Chi-Square test; p=0.514). All systemic reactions were mild or moderate; the most commonly reported events were malaise, headache, myalgia.

**Unsolicited adverse events**

Within 28 days of any vaccination there were 146 unsolicited AEs among 52 volunteers, of which 92 (63%) AEs were mild and 54 (37%) moderate. One hundred and twelve (112) AEs were reported by 42 vaccinees and 34 by 10 placebo recipients (Randomization: Vaccine : Placebo = 4:1).

During the entire study, 63 of 75 volunteers reported a total of 209 non-serious unsolicited adverse events (AEs), of which 134 (64.1%) were mild (Grade 1) and 75 (35.9%) moderate (Grade 2). None were assessed as severe (Grade 3). One hundred and sixty (160) AEs were reported from vaccinees of all groups and 49 AEs from placebo recipients. Six (2.9%) of the 209 AEs were judged to be possibly related to the investigational product, none probably or definitely related. One event (dizziness) was graded as moderate, the remaining 5 events (vasovagal response (n=2), dizziness, aphthous stomatitis and thoracic pain) were mild. Two of the 6 AEs judged to be related to investigational product (IP) occurred in placebo recipients (mild thoracic pain, mild dizziness). The 4 related AEs in vaccinees occurred on the day of vaccination (2 vasovagal responses, both in Group 2), on day 1 (dizziness in Group 1) and on day 3 (mild aphthous stomatitis in Group 5) after the 1^st^ vaccination timepoint. The events assessed as related in placebo (normal saline) recipients occurred one on the day of the 2^nd^ IM/EP administration (thoracic pain) and the other on day 1 after saline given IM (dizziness), respectively.

Overall, 47% vaccine and 53% placebo recipients reported a moderate AE (Chi-square test; p=0.644). In Groups 1-3, 50% (18/36) vaccine and 44% (4/9) placebo recipients reported moderate adverse events (p=1.000, Fisher’s exact 2-tailed test). In Groups 4-5, 58% (14/24) vaccine and 50% (3/6) placebo recipients reported moderate adverse events (p=1.000, Fisher’s exact 2-tailed test).

**Laboratory abnormalities**

No severe abnormal hematology and no moderate or severe chemistry abnormalities were reported. Mildly abnormal chemistry results were observed in four volunteers, none of which was assessed as clinically significant by the principal investigator at the clinical site.

Moderate abnormal hematology results were observed in nine volunteers (distributed across all 5 Groups), none of which was assessed as clinically significant. The most common hematological abnormality was absolute neutropenia (observed in 6 vaccine and 1 placebo recipient), followed by decrease in hemoglobin (in 2 vaccine recipients) and thrombocytopenia (in 1 vaccine recipient). All abnormal values improved, returned to normal range or to pre-vaccination levels during follow-up. No abnormal laboratory result was reported as adverse event (data not shown).
