# Supplementary material for: A Phase I Double Blind, Placebo-Controlled, Randomized Study of the Safety and Immunogenicity of Electroporated HIV DNA with or without Interleukin 12 in Prime-Boost Combinations with an Ad35 HIV Vaccine in Healthy HIV-Seronegative African Adults
Source: PLoS One. 2015 Aug 7;10(8):e0134287. doi: 10.1371/journal.pone.0134287 (PMC4529153; doi:10.1371/journal.pone.0134287)
Supplement: S1 Table — (DOCX) [file pone.0134287.s004.docx]

**S1 Table SUMMARY OF ADVERSE EVENTS WITHIN 28 DAYS OF ANY VACCINATION: BY RELATIONSHIP, SEVERITY AND STUDY GROUP**

|  | **Frequency and percent (% of total number volunteers per study arm/treatment)** | | | | | | | |
| --- | --- | --- | --- | --- | --- | --- | --- | --- |
|  | **Group 1** | **Group 2** | **Group 3** | **Group 4** | **Group 5** | **All Groups** | | |
|  | **HIVMAG /Ad35 N=12** | **HIVMAG+ IL-12**  **(100μg) /Ad35 N=12** | **HIVMAG+ IL-12 (1000μg) /Ad35 N=12** | **HIVMAG+ IL-12 (1000μg) /Ad35 N=12** | **Ad35 /HIVMAG+ IL-12 (1000μg) N=12** | **Placebo N=15** | **Vaccine N=60** | **Total N=75** |
| **Any** | | | | | | | | |
| Grade 1 | 3 (25.0) | 4 (33.3) | 5 (41.7) | 4 (33.3) | 3 (25.0) | 4 (26.7) | 19 (31.7) | 23 (30.7) |
| Grade 2 | 6 (50.0) | 5 (41.7) | 6 (50.0) | 2 (16.7) | 4 (33.3) | 6 (40.0) | 23 (38.3) | 29 (38.7) |
| **None** | | | | | | | | |
| Grade 1 | 4 (33.3) | 4 (33.3) | 5 (41.7) | 4 (33.3) | 2 (16.7) | 5 (33.3) | 19 (31.7) | 24 (32.0) |
| Grade 2 | 5 (41.7) | 5 (41.7) | 4 (33.3) | 1 (8.3) | 4 (33.3) | 5 (33.3) | 19 (31.7) | 24 (32.0) |
| **Unlikely** | | | | | | | | |
| Grade 1 | 3 (25.0) | 0 (0.0) | 3 (25.0) | 1 (8.3) | 2 (16.7) | 4 (26.7) | 9 (15.0) | 13 (17.3) |
| Grade 2 | 0 (0.0) | 1 (8.3) | 2 (16.7) | 1 (8.3) | 0 (0.0) | 1 (6.7) | 4 (6.7) | 5 (6.7) |
| **Possible** | | | | | | | | |
| Grade 1 | 0 (0.0) | 2 (16.7) | 0 (0.0) | 0 (0.0) | 1 (8.3) | 2 (13.3) | 3 (5.0) | 5 (6.7) |
| Grade 2 | 1 (8.3) | 0 (0.0) | 0 (0.0) | 0 (0.0) | 0 (0.0) | 0 (0.0) | 1 (1.7) | 1 (1.3) |
