# Supplementary material for: A Phase I Double Blind, Placebo-Controlled, Randomized Study of the Safety and Immunogenicity of Electroporated HIV DNA with or without Interleukin 12 in Prime-Boost Combinations with an Ad35 HIV Vaccine in Healthy HIV-Seronegative African Adults
Source: PLoS One. 2015 Aug 7;10(8):e0134287. doi: 10.1371/journal.pone.0134287 (PMC4529153; doi:10.1371/journal.pone.0134287)
Supplement: S2 Table — (DOCX) [file pone.0134287.s005.docx]

**S2 Table. SUMMARY OF EP TOLERABILITY ASSESSMENTS OVERALL**

| **Assessment Time Relative to EP. Number and percent (%)** | | | | |
| --- | --- | --- | --- | --- |
| **Maximum Rating per Volunteer** | **Before** | **Immediately after** | **After 10 min** | **After 30 min** |
| **None** | 19 (25.3) | 1 (1.3) | 10 (13.3) | 7 (9.3) |
| **Light** | 42 (56.0) | 30 (40.0) | 40 (53.3) | 45 (60.0) |
| **Uncomfortable** | 14 (18.7) | 35 (46.7) | 22 (29.3) | 21 (28.0) |
| **Intense** | 0 | 9 (12.0) | 3 (4.0) | 2 (2.7) |
| **Severe** | 0 | 0 | 0 | 0 |
| **Very Severe** | 0 | 0 | 0 | 0 |
