# Supplementary material for: A Phase I Double Blind, Placebo-Controlled, Randomized Study of the Safety and Immunogenicity of Electroporated HIV DNA with or without Interleukin 12 in Prime-Boost Combinations with an Ad35 HIV Vaccine in Healthy HIV-Seronegative African Adults
Source: PLoS One. 2015 Aug 7;10(8):e0134287. doi: 10.1371/journal.pone.0134287 (PMC4529153; doi:10.1371/journal.pone.0134287)
Supplement: S3 Table — (DOCX) [file pone.0134287.s006.docx]

**S3 Table. Multivariate Proportional Odds Regression Analyses of Tolerance**

(separate analyses were done for each time point and p-values are unadjusted)

Pain was reported on a scale of 0 - 5 (none, light, uncomfortable, intense, severe, very severe).

|  | **Odds of higher pain** | **Wald 95% CI** | |  |
| --- | --- | --- | --- | --- |
|  |  | **Lower** | **Upper** | **p-value** |
| **Before EP** | | | | |
| Age | 0.991 | 0.920 | 1.067 | 0.8103 |
| Females ^1^ | 1.699 | 0.580 | 4.976 | 0.3338 |
| HIV-MAG ^2^ | 4.191 | 0.900 | 19.520 | 0.0679 |
| HIV-MAG + IL-12 (100mcg) ^2^ | 2.646 | 0.550 | 12.739 | 0.2248 |
| HIV-MAG + IL-12 (1000mcg) ^2^ | 1.213 | 0.368 | 3.995 | 0.7512 |
| BMI | 0.982 | 0.858 | 1.125 | 0.7965 |
| Skin-fold thickness | 0.973 | 0.880 | 1.077 | 0.6025 |
| **At EP** | | | | |
| Age | 0.970 | 0.899 | 1.047 | 0.4295 |
| Females ^1^ | 0.841 | 0.286 | 2.469 | 0.7521 |
| HIV-MAG ^2^ | 2.225 | 0.498 | 9.935 | 0.2948 |
| HIV-MAG + IL-12 (100mcg) ^2^ | 0.857 | 0.181 | 4.054 | 0.8459 |
| HIV-MAG + IL-12 (1000mcg) ^2^ | 0.883 | 0.270 | 2.884 | 0.8365 |
| BMI | 0.828 | 0.710 | 0.966 | **0.0166** |
| Skin-fold thickness | 1.086 | 0.977 | 1.209 | 0.1274 |
| **After 10 minutes** | | | | |
| Age | 0.927 | 0.859 | 1.000 | 0.0514 |
| Females ^1^ | 1.889 | 0.642 | 5.561 | 0.2483 |
| HIV-MAG ^2^ | 1.880 | 0.418 | 8.456 | 0.4105 |
| HIV-MAG + IL-12 (100mcg) ^2^ | 5.056 | 1.035 | 24.698 | **0.0452** |
| HIV-MAG + IL-12 (1000mcg) ^2^ | 0.957 | 0.287 | 3.187 | 0.9430 |
| BMI | 0.984 | 0.859 | 1.126 | 0.8110 |
| Skin-fold thickness | 1.026 | 0.927 | 1.136 | 0.6221 |
| **After 30 minutes** | | | | |
| Age | 0.895 | 0.820 | 0.977 | **0.0128** |
| Females ^1^ | 11.143 | 3.006 | 41.311 | **0.0003** |
| HIV-MAG ^2^ | 10.062 | 1.693 | 59.799 | **0.0111** |
| HIV-MAG + IL-12 (100mcg) ^2^ | 15.413 | 2.517 | 94.390 | **0.0031** |
| HIV-MAG + IL-12 (1000mcg) ^2^ | 3.246 | 0.796 | 13.234 | 0.1005 |
| BMI | 0.769 | 0.651 | 0.909 | **0.0020** |
| Skin-fold thickness | 1.112 | 0.986 | 1.254 | 0.0829 |
| ^1^  Females Versus Males.    ^2^  Treatment Versus Placebo | | | | |
