# Supplementary material for: A Phase I Double Blind, Placebo-Controlled, Randomized Study of the Safety and Immunogenicity of Electroporated HIV DNA with or without Interleukin 12 in Prime-Boost Combinations with an Ad35 HIV Vaccine in Healthy HIV-Seronegative African Adults
Source: PLoS One. 2015 Aug 7;10(8):e0134287. doi: 10.1371/journal.pone.0134287 (PMC4529153; doi:10.1371/journal.pone.0134287)
Supplement: S4 Table — (DOCX) [file pone.0134287.s007.docx]

**S4. Table. Pair-wise comparisons of ELISpot responses 2 weeks after the final vaccination**

|  | **Arm 2** | **Arm 3** | **Arm 4** | **Arm 5** | **Placebo** |
| --- | --- | --- | --- | --- | --- |
| **Volunteers responding to any antigen** | | | | | |
|  | **(9/11, 81.8%)** | **(8/9, 88.9%)** | **(5/10, 50%)** | **(5/11, 45.5%)** | **(2/12, 16.7%)** |
| **Arm 1 (8/11, 72.7%)** | 1.0 (0.216) | 0.591 (0.445) | 0.387 (**0.016**) | 0.387 (**0.011**) | 0.012 (<.001) |
| **Arm 2 (9/11, 81.8%)** |  | 1.0 (0.729) | 0.183 (0.071) | 0.183 (**0.041**) | 0.003 (<.001) |
| **Arm 3 (8/9, 88.9%)** |  |  | 0.141 (0.068) | 0.070 (0.054) | 0.002 (<.001) |
| **Arm 4 (5/10, 50%)** |  |  |  | 1.0 (0.786) | 0.172 (0.012) |
| **Arm 5 (5/11, 45.5%)** |  |  |  |  | 0.193 (<.001) |
| **Volunteers responding to any Ad35-GRIN/ENV antigen** | | | | | |
|  | **(8/11, 72.7%)** | **(8/9, 88.9%)** | **(4/10, 40%)** | **(5/11, 45.5%)** | **(0/12)** |
| **Arm 1 (8/11, 72.7%)** | 1.0 (0.072) | 0.591 (0. 340) | 0.198 (**0.015**) | 0.387 (**0.012**) | 0.0003 (<.001) |
| **Arm 2 (8/11, 72.7%)** |  | 0.591 (0.431) | 0.198 (0.256) | 0.387 (0.366) | 0.0003 (<.001) |
| **Arm 3 (8/9, 88.9%)** |  |  | 0.057 (0.105) | 0.070 (0.119) | <.0001 (<.001) |
| **Arm 4 (4/10, 40%)** |  |  |  | 1.0 (0.700) | 0.029 (0.004) |
| **Arm 5 (5/11, 45.5%)** |  |  |  |  | 0.014 (<.001) |
| **Volunteers responding to any HIV-MAG antigen** | | | | | |
|  | **(8/11, 72.7%)** | **(8/9, 88.9%)** | **(4/10, 40%)** | **(3/11, 27.3%)** | **(2/12, 16.7%)** |
| **Arm 1 (8/11, 72.7%)** | 1.0 (0.603) | 0.591 (0.613) | 0.198 (**0.038**) | 0.086 (**0.019**) | 0.012 (<.001) |
| **Arm 2 (8/11, 72.7%)** |  | 0.591 (0.965) | 0.198 (**0.041**) | 0.086 (**0.017**) | 0.012 (<.001) |
| **Arm 3 (8/9, 88.9%)** |  |  | 0.057 (0.066) | 0.010 (**0.031**) | 0.002 (<.001) |
| **Arm 4 (4/10, 40%)** |  |  |  | 0.659 (0.963) | 0.348 (0.023) |
| **Arm 5 (3/11, 27.3%)** |  |  |  |  | 0.640 (0.011) |

Each cell contains two p-values. The first is a comparison of the proportion of responders and is based on Fisher’s exact 2-tailed test, the second (in parentheses) is a comparison of the median response per volunteer based on Student’s t-test of the natural log transform (responses<1 were replaced with 1). The p-values are not adjusted for multiple comparisons.
