# Supplementary material for: A Phase I Double Blind, Placebo-Controlled, Randomized Study of the Safety and Immunogenicity of Electroporated HIV DNA with or without Interleukin 12 in Prime-Boost Combinations with an Ad35 HIV Vaccine in Healthy HIV-Seronegative African Adults
Source: PLoS One. 2015 Aug 7;10(8):e0134287. doi: 10.1371/journal.pone.0134287 (PMC4529153; doi:10.1371/journal.pone.0134287)
Supplement: S5 Table — (DOCX) [file pone.0134287.s008.docx]

**S5. Table. Env and Gag ELISA titers and response rates**

|  |  | Env A* |  | Env B* |  | Gag |  |
| --- | --- | --- | --- | --- | --- | --- | --- |
|  | Week | % responders | GMT** | %  responders | GMT** | %  responders | GMT** |
| Group 1 | Baseline | 0 |  | 0 |  | 25 | 171 |
|  | 4 Weeks  Post-Vac.3 | 0 |  | 0 |  | 25 | 100 |
|  | 2 Weeks  Post-Vac.4 | 89 | 334 | 56 | 362 | 44.4 | 150 |
| Group 2 | Baseline | 0 |  | 0 |  | 8.3 | 100 |
|  | 4 Weeks  Post-Vac.3 | 0 |  | 0 |  | 8.3 | 100 |
|  | 2 Weeks  Post-Vac.4 | 89 | 224 | 78 | 397 | 11.1 | 100 |
| Group 3 | Baseline | 0 |  | 0 |  | 0 |  |
|  | 4 Weeks  Post-Vac.3 | 0 |  | 0 |  | 8.3 | 500 |
|  | 2 Weeks  Post-Vac.4 | 67 | 224 | 44.4 | 224 | 11 | 500 |
| Group 4 | Baseline | 0 |  | 8.3 | 100 | 0 |  |
|  | 4 Weeks  Post-Vac.1 | 0 |  | 0 |  | 0 |  |
|  | 2 Weeks  Post-Vac.2 | 25 | 100 | 8.3 | 100 | 0 |  |
| Group 5 | Baseline | 0 |  | 0 |  | 17 | 100 |
|  | 4 Weeks  Post-Vac.1 | 25 | 171 | 8.3 | 500 | 33 | 100 |
|  | 2 Weeks  Post-Vac.2 | 25 | 100 | 8.3 | 100 | 33 | 100 |

*Env A = Env subtype A, UG37 gp140 protein and Env B = Env subtype B, 6101 gp140 protein (70% identity).

**GMT = geometric mean titer
