# Supplementary material for: A Phase I Double Blind, Placebo-Controlled, Randomized Study of the Safety and Immunogenicity of Electroporated HIV DNA with or without Interleukin 12 in Prime-Boost Combinations with an Ad35 HIV Vaccine in Healthy HIV-Seronegative African Adults
Source: PLoS One. 2015 Aug 7;10(8):e0134287. doi: 10.1371/journal.pone.0134287 (PMC4529153; doi:10.1371/journal.pone.0134287)
Supplement: S2 Fig — (DOCX) [file pone.0134287.s010.docx]

**S2 Fig. ICS gating strategy**

**
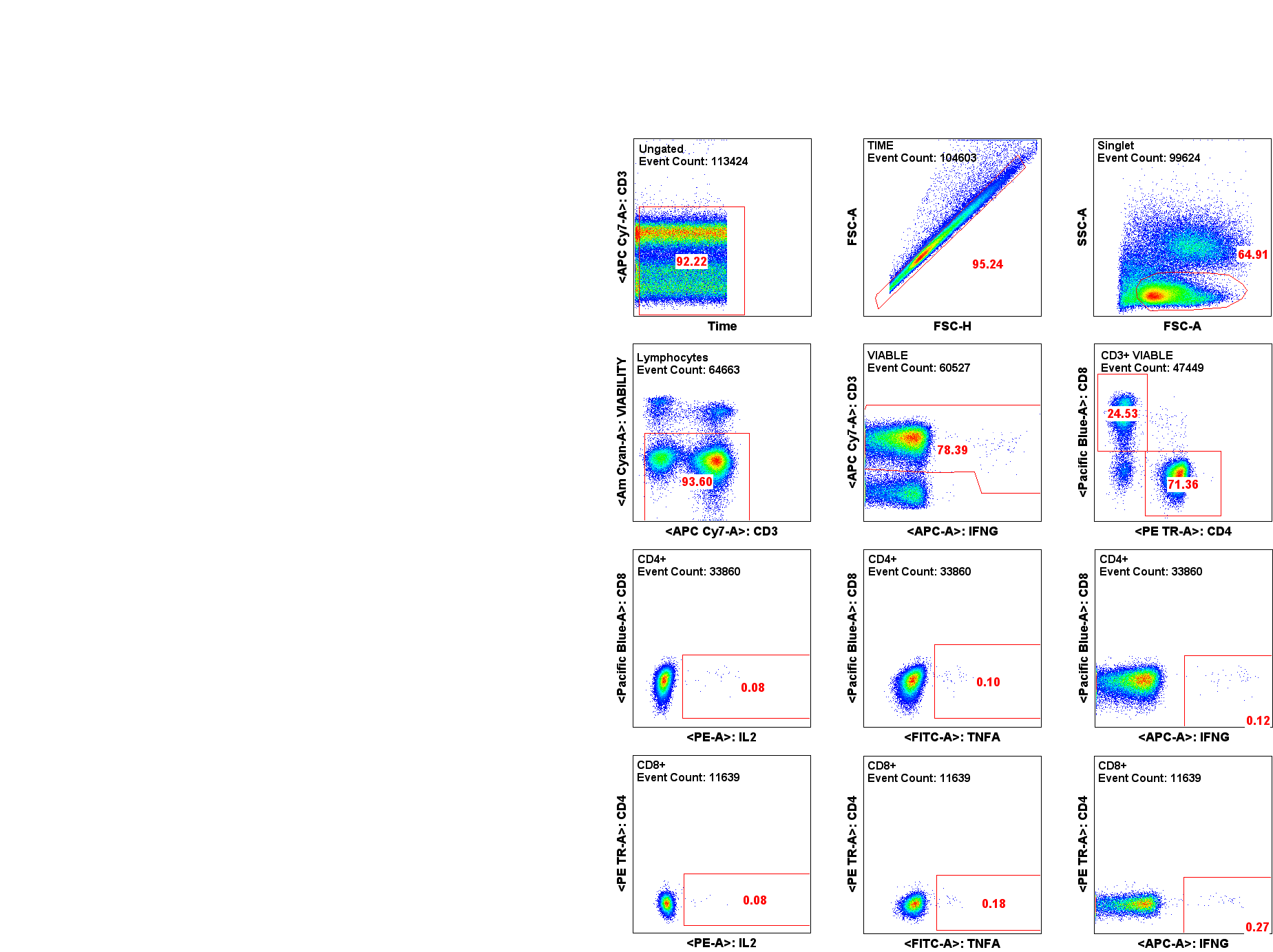
**

**S2 Fig.** Representative ICS plots describing gating strategy used for measuring cytokines following antigen stimulation. Example shows a PBMCs following CMV pp65 peptide stimulation.
