# Supplementary material for: A Phase I Double Blind, Placebo-Controlled, Randomized Study of the Safety and Immunogenicity of Electroporated HIV DNA with or without Interleukin 12 in Prime-Boost Combinations with an Ad35 HIV Vaccine in Healthy HIV-Seronegative African Adults
Source: PLoS One. 2015 Aug 7;10(8):e0134287. doi: 10.1371/journal.pone.0134287 (PMC4529153; doi:10.1371/journal.pone.0134287)
Supplement: S3 Fig — (DOCX) [file pone.0134287.s011.docx]

**S3 Figure. Viral Inhibition Assay Responses post Prime and Boost**

**Log VIA Inhibition**

**S3 Fig. Viral Inhibition Assay.** VIA was assessed 2-4 weeks post HIVMAG (x3) (Figure S3, top panel) and at 2 weeks post Ad35-GRIN/Env boost (Figure S3, bottom panel). Pink: Placebo, Blue: Group 1 Vac., Red: Group 2 Vac., Green: Group 3 Vac. The data is shown as log inhibition each box and whisker plot summarizes the positive responses (i.e., the median, 1st and 3rd quartiles and minimum/maximum). The dashed line shows the VIA cut-off which is defined by additional criteria in the materials and methods. Table 4 in the manuscript shows the descriptive statistics for VIA.
